# Development, validation, and assessment of a reliable and white method for the analysis of water-based perfumes by pipette-tip micro-solid-phase extraction combined with dual detection gas chromatography

**DOI:** 10.1007/s00216-025-05889-x

**Published:** 2025-05-07

**Authors:** Gaia Bechis, Giulia Quaranta, Carlo Bicchi, Arianna Marengo, Barbara Sgorbini, Patrizia Rubiolo, Cecilia Cagliero

**Affiliations:** Department of Drug Science and Technology, University of Turin, Via Pietro Giuria 9, 10125 Turin, Italy

**Keywords:** Aqueous fragrances, Pipette-tip micro-solid-phase extraction, Method evaluation metric tools, Gas chromatography, Green analytical chemistry

## Abstract

The analysis of possible allergens, impurities, and contaminants in perfumes and the determination of their volatile fingerprints are of high importance in determining their quality. At the same time, the analysis of perfumes with a significant water content can require complex procedures or large amounts of material. These new products are becoming increasingly popular as they reduce skin dryness and irritation, but they require the addition of additives to improve the solubility of all fragrance components. Direct injection into gas chromatography can lead to interferences and requires more frequent maintenance of the chromatographic system, while traditional extraction methods need several steps, a large volume of sample and material, and the use of toxic solvents. In this study, pipette-tip micro-solid-phase extraction (PT-µSPE) was used and optimized as an innovative, simple, sustainable, and time-saving approach. It requires only 10 mg Celite and 10 mg sample while elution is carried out with 100 mg heptane/ethyl acetate (70/30, p/p). The extraction is coupled with GC analysis with simultaneous FID and MS detection (GC–MS/FID), which is used for qualitative, semi-quantitative, and quantitative purposes. The performance of the PT-µSPE method was validated and successfully applied to commercial samples. In addition, the performance of the developed method and of the reference approaches was evaluated, focusing mainly on the overall impact of analysis in accordance with the principles of White Analytical Chemistry. The results show that the PT-µSPE method is more balanced than the conventional methods in terms of analytical performance, greenness, and practical efficiency, making it a valuable alternative for routine use in the fragrance industry.

**Graphical Abstract:**

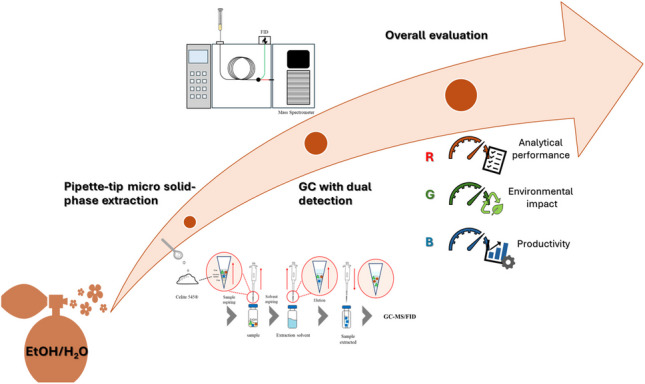

**Supplementary Information:**

The online version contains supplementary material available at 10.1007/s00216-025-05889-x.

## Introduction

Analyses in the fragrance field can cover a wide range of topics, from the qualitative and semi-quantitative evaluation of the chemical fingerprint of the fragrance to the quantitative determination of target substances regulated by international organizations. In this sense, particular attention is paid to the extended list of potential “fragrance allergens” listed in Annex III of Regulation (EC) No 1223/2009 [1], as amended by Commission Regulation (EU) 2023/1545 [2], based on the opinion of the Scientific Committee on Consumer Safety (SCCS). Several analytical methods have been developed for the analysis, identification, and quantification of these compounds in cosmetics [3–5] with the UNI EN 16274:2021 standard method considered as a consensus method for fragrance quality control laboratories [6]. Fragrance allergens must be declared in the list of ingredients of the final product, according to the above-mentioned regulations, if their concentration exceeds 0.001% w/w in leave-on products and 0.01% w/w in rinse-off products. Sample preparation is usually not required for the analysis of perfumes, as the high ethanol content allows complete solubilization of the fragrance concentrate (mixture of fragrance raw materials) in the final product and allows direct injection into the gas chromatography system coupled with mass spectrometry (GC–MS) and/or with flame ionization detector (GC-FID). Nevertheless, cosmetic companies are starting to produce and market fragrances with a lower alcohol content to avoid skin dryness or irritation. These perfumes with higher water content require the addition of cosmetic agents (such as stabilizers, emulsifiers, homogenizers) to obtain optimal sensoriality. The direct injection of this type of products can lead to instrumental problems related to the direct injection of high amount of water [7–9] and the incomplete volatility of the cosmetic additives. Therefore, pretreatment of the samples may be necessary. Solid-phase extraction (SPE) methods developed for the determination of volatile compounds from complex food matrices can be used, although they are generally time consuming and requires a large amount of solvents, materials, and toxic reagents [10–13]. Alternatively, disposable extraction devices consisting of PDMS foam cylinders have been introduced to determine fragrance ingredients in aqueous surfactant solutions [14]. However, they require a thermodesorption step of the devices to recover sampled analytes before GC–MS analysis, thus requiring a dedicated instrumentation. These limitations can influence the adoption of a method for the laboratories, as they have to face not only cost and productivity, but also increasing regulatory and public attention to environmental sustainability.

Following the principles of Green Analytical Chemistry (GAC) [15], different approaches have been developed, mainly based on miniaturization, automation, and use of renewable resources, to reduce the environmental impact of analysis and sample preparation [16]. Within this tendency, pipette-tip micro-solid-phase extraction (PT-µSPE) can be an interesting approach. It consists in confining a small amount of sorbent material at the bottom of a pipette tip and simply aspirating and dispensing sample, washing (if required) and elution solutions [17–20]. This approach is very versatile (as sorbent and solvents can be optimized depending on the application), is easy to automate, and requires only a small amount of materials. It could therefore be defined a “green” approach, but a reliable assessment of its greenness is crucial.

In recent years, several green metrics have been developed to measure the “greenness” of an analytical process [21]. They can guide chemists towards more sustainable practices, although each tool applies its own calculations and criteria and, with the exception of the life cycle assessment (LCA) approach [22], they do not allow to quantitatively and objectively estimate the impact on the environment (for instance in terms of global warming). Moreover, most tools consider only one criterion (greenness or productivity), while it would be beneficial for laboratories that have to choose an analytical method, to evaluate its overall performance. Indeed, the Red–Green–Blue model was developed to evaluate the three primary parameters of each analytical method [23]. The red section stands for the analytical performance of a method, which is usually evaluated by a validation process. The green section stands for the safety and environmental friendliness of an analytical method, taking into account the GAC principles. Finally, the blue section concerns productivity and practical effectiveness. The variables for each primary attribute are selected by the user depending on the specifications of the method, the intended application, and user’s needs. The RGB model inspired the concepts behind White Analytical Chemistry (WAC) [24], which was introduced in 2021 as an extension of GAC and contains a list of the specific criteria that should be adopted for each of the three attributes. This approach has recently been simplified by the development of a new and user-friendly version called RGBfast, which further reduces the number of parameters to be considered and is therefore of more general use [25]. At the same time, Kiwfo et al. [26] developed a new index to extend the global assessment of a method: the Need, Quality, and Sustainability (NQS) index. The NQS index evaluates the analytical method in terms of the “need” of the chosen application [27], the “quality” (according to the WAC principles) and the “sustainability” (in line with the Sustainable Development Goals, SDGs) [28]. The NQS index of analytical chemistry is represented by a triangular pyramid whose height and shape varies or is distorted depending on the performance of the three attributes.

Based on the above considerations, this study aims to develop a method that can be applied to characterize perfumes with a significant water content. Both the fragrance fingerprint and the amount of selected target markers were evaluated by removing interfering water and cosmetic agents without affecting the qualitative composition of the sample. Starting from a reference sample preparation method commonly used for the extraction of apolar volatile compounds from hydroalcoholic samples, a micro-solid-phase extraction method was developed by optimizing the extraction solvent and sorbent and their amounts, as well as the number of individual steps, and the extraction time. The optimized pipette-tip micro-solid-phase extraction (PT-µSPE) was validated and compared to conventional approaches used in cosmetic quality control laboratories in terms of greenness and overall method performance.

## Experimental

### Chemicals and materials

Cyclohexane (99.9% purity), heptane (96% purity), acetonitrile (99.9% purity), and ethyl acetate (99.5% purity) were purchased from Merck (Milan, Italy) and used as extraction solvents. Ethanol absolute (99.9% purity) from Merck (Milan, Italy) and ultrapure water with a resistivity of 18.2 MὨ cm obtained from a Milli-Q water purification system (Bedford, MA, USA) were used as diluents. Celite 545^®^ and EXtrelut^®^ NT from Merck (Milan, Italy) were used as sorbent materials for the extraction methods.

Stock solutions of common fragrance compounds (see Table S1) were prepared by diluting 50.0 mg of the analyte in 5 g of ethanol (10 g kg^−1^). These solutions were then used to prepare the following mixtures, which were diluted to a concentration of 1.0 g kg^−1^ in ethanol: Mixture 1 consisted of benzaldehyde (95.3% purity) (CAS 100–52-7), limonene (98.4% purity) (CAS 138–86-3), ethyl linalool (including isomer 1 and isomer 2 with 38.6% and 60.6% of purity, respectively) (CAS 10339–55-6), coumarin (99.2%) (CAS 91–64-5), tetramethyl acetyloctahydronaphthalenes (including isomers 1, 2, and 3 at 75.3%, 9.3%, and 14.5% purity, respectively) (CAS 54464–57-2), ambroxide (99.0% purity) (CAS 6790–58-5), and 1,3,4,6,7,8-hexahydro-4,6,6,7,8,8-hexamethylcyclopenta-γ−2-benzopyran (97.8% purity) (CAS 1222–05-5). On the other hand, linalyl acetate (72.1% purity) (CAS 115–95-7), *trans*-β-caryophyllene (98.1% purity) (CAS 87–44-5), methylenedioxyphenyl methylpropanal (95.0% purity) (CAS 1205–17-0), benzyl benzoate (99.9% purity) (CAS 120–51-4), oxacyclohexadecenone (12E) (including isomers 1, 2, and 3 with 40.6%, 28.1%, and 27.4% purity, respectively) (CAS 111879–80-2), and oxacycloheptadec-10-en-2-one (99.9% purity) (CAS 28645–51-4) were selected as target analytes for Mixture 2. All standards were from the author’s collection; their supplier is reported in Table S1. In addition, 1,4-dibromobenzene (99.7% purity) (CAS 106–37-6) and 4,4′-dibromobiphenyl (99.7% purity) (CAS 92–86-4), purchased from Merck (Milan, Italy), were added to the working standard solutions and the real samples as internal standards at a concentration of 50 mg kg^−1^. The purity of the selected target analytes, indicated by the supplier, was confirmed by GC-FID analysis of each compound diluted in ethanol at a concentration of 100 mg kg^−1^ under the conditions described in section “Analysis conditions”. Eight commercial perfumes, consisting of different formulations, in terms of components and their relative amount, and containing glycerin and PEG-40 hydrogenated castor oil as cosmetic agents, were analyzed. Their composition is reported in Table S2. For method validation, Perfume 5 and Perfume 6 were spiked with increasing concentrations (n = 3) of standard mixtures 1 and 2 to create a calibration curve in the range 1–500 mg kg^−1^. After addition of the internal standards, all the samples were analyzed by direct injection and after extraction, while the spiked samples were analyzed only after extraction.

The final extract obtained after extractions, with the exception of PT-µSPE (see section “Overall evaluation of the method”), was filtered using a 0.20-μm PVDF filter (CPS Analytica, Milan, Italy) prior to injection into the GC–MS/FID system. The micropipettes and the tips used for PT-µSPE were also supplied by CPS Analytica (Milan, Italy).

### Instruments and equipment

GC–MS/FID analyses were performed using a Shimadzu GC–MS/FID system consisting of a Shimadzu GC2010 gas chromatograph coupled to an FID detector and a Shimadzu QP2010 Plus mass spectrometer (Shimadzu, Milan, Italy). A MultiPurpose Autoinjector AOC 5000 (Shimadzu, Milan, Italy) was used as an autosampler. Energy consumption was measured with a Zhurui PR10 Power Meter Plug (Zhurui, China). A centrifuge (model 5702 from Eppendorf, Hamburg, Germany), a vortex mixer (ArgoLab, Modena, Italy), and an analytical balance with a minimum readability of 0.01 mg (Sartorius, Madrid, Spain) were used.

### Analysis conditions

Samples were analyzed using a MEGA-5MS (20 m length × 0.18 mm *d*_*c*_, 0.50 µm *d*_*f*_) capillary column (MEGA, Milan, Italy). Data were collected simultaneously from both the MS and FID detectors. A passive Y-shaped glass splitter (MEGA, Milan, Italy) was attached to the outlet of the column and connected to two deactivated capillary column segments (MEGA, Milan, Italy). The Effluent Splitter Pressure Calculator, supplied by Agilent (Milan, Italy), was used to determine the conditions governing the flow ratio between the two detectors. The inlet pressure was kept constant to avoid discrimination during the temperature program. A deactivated fused silica capillary column of 2 m length × 0.18 mm *d*_*c*_ was connected to the FID detector, and another of 1 m length × 0.10 mm *d*_*c*_ was connected to the MS detector. MS analysis was used for identification and SIM quantification by a calibration curve of investigated analytes and data from FID detection were employed to determine the relative percent composition.

The GC analysis conditions were as follows: injection temperature, 220 °C; carrier gas, helium; constant pressure of 172.4 kPa; injection mode, split; split ratio, 10:1; injection volume, 1 μL. The oven temperature program was from 50 °C (hold time 0.62 min) to 250 °C at 4 °C min^−1^. The solvent delay time was 3 min, and the carrier gas saver was set with a split ratio of 5 after 5.0 min.

The operative MS conditions were as follows: transfer line, 230 °C; ion source temperature, 200 °C. The MS operated in electron impact (EI) ionization mode at 70 eV and acquired simultaneously in SIM and scan mode with a scan rate of 666 u/s and a mass range of 35–350 m/z. The ions selected for SIM acquisition for each compound are listed in Table S1. After injection of Perfumes 2 and 6, some analytes of interest, i.e., suspected allergens and representative fragrance ingredients (Table S1) characterized by different physicochemical properties and belonging to different chemical classes, were selected for further investigation (see section “Development of simultaneous quantitative and semi-quantitative chromatographic method”). Identification was performed by spectral similarity matching estimated from commercial and proprietary databases and by *I*^T^ comparison with commercial databases of retention indexes. Working solutions of the two standard mixtures were prepared by diluting the stock solution with ethanol at different concentrations (from 0.2 mg kg^−1^ to 500 mg kg^−1^) and used for quantification. Calibration curves were generated by normalizing the peak areas to those of the internal standards.

The FID conditions were as follows: the detector temperature was maintained at 250 °C; make-up gas flow (N_2_) was 60 mL min^−1^; H_2_ flow, 40 mL min^−1^; and air flow, 450 mL min^−1^. The FID sampling rate was 40 ms.

### Extraction methods

**Fig. 1:**
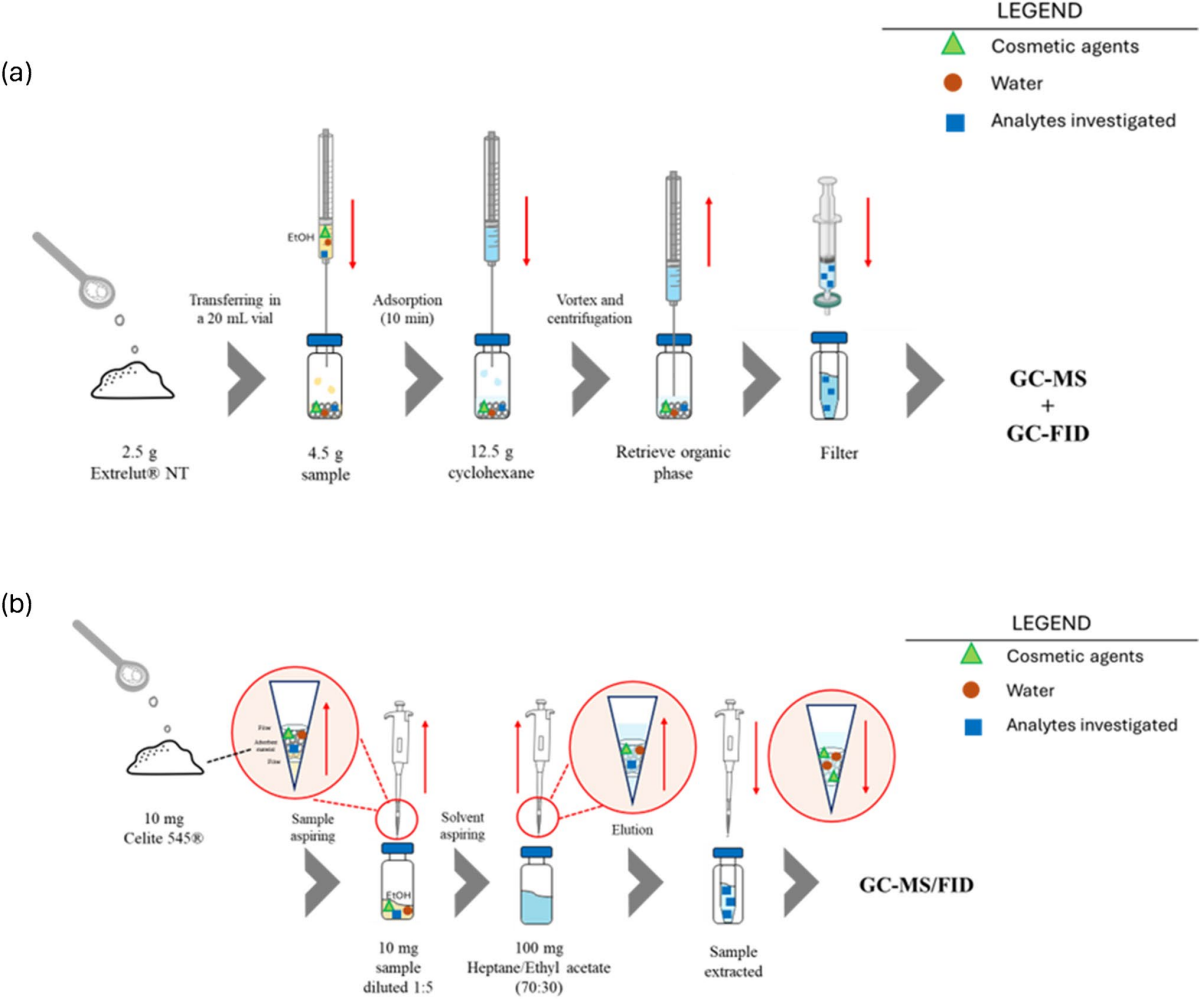
Schematic diagram of the reference extraction method (**a**) and pipette-tip micro-solid-phase extraction (PT-µSPE) method (**b**)

A method developed for the determination of aroma components in sweet sparkling wine was used as a reference approach to extract fragrance components from the investigated perfumes [29, 30]. In particular, 4.5 g of the sample and 2.5 g of EXtrelut^®^ NT, used as sorbent material, were thoroughly mixed with a spatula to ensure that the sample was completely adsorbed. Then 12.5 g of cyclohexane was added as eluent and vortexed three times for 2 min. After centrifugation at 4000 rpm for 5 min, the organic phase was retrieved, filtered with a 0.20-μm PVDF filter, collected in a vial, and injected into the GC–MS/FID system. Figure 1a shows this method defined “reference method.”

Based on the reference method, the sample preparation conditions were optimized in terms of extraction solvent (heptane, cyclohexane, ethyl acetate, acetonitrile, and mixtures of them) and sorbent (EXtrelut^®^ and Celite 545^®^) as well as amount of sample, sorbent, and solvent.

Subsequently, the pipette-tip micro-solid-phase extraction (PT-µSPE) configuration was tested to reduce the number of steps required for extraction. The optimized procedure was as follows: 10 mg of Celite 545^®^ was weighed and packed into a 1000 µL pipette tip, using two small pieces of paper filter at either end of the pipette tip to avoid loss of the sorbent phase. Ten milligrams of the sample, previously diluted 1:5 with ethanol, were then weighed into a 4 mL vial and slowly aspirated into the tip of the micropipette. This procedure allows the sample to be absorbed into the Celite. The analytes were eluted by aspirating 100 mg of heptane/ethyl acetate (70/30, p/p) previously weighed into the same vial and dispensed back through the absorbent material. The extracted analytes were collected in a 2 mL vial with a restrictor for direct GC–MS/FID analysis. The process is summarized in Fig. 1b.

### Validation of the method

The proposed PT-µSPE method was validated in accordance with Regulation (EU) 2021/808 [31]. The linearity of the analytical response was evaluated by calculating the coefficient of determination of the calibration curves (*R*^2^) obtained by analyzing the two standard mixtures after direct injection and after the extraction process at different concentrations, as previously described. Quantification was performed in selected ion monitoring (SIM) using specific ions for each target compound, as indicated in Table S1. Relative percentage composition determination (from here semi-quantification) was performed by GC-FID analysis. LOD and LOQ were experimentally determined by decreasing the concentration of analytes injected or subjected to extraction until a signal-to-noise ratio (S/N) of 3 and 10, respectively [32].

Repeatability, expressed as RSD%, was estimated from three independent extractions analyzed in triplicate (*n* = 9).

Reproducibility (intermediate precision) was estimated under intra-laboratory conditions by calculating the mean repeatability on the three repeated extractions carried out by different operators in three different days and analyzed in triplicate (*n* = 27).

Measurement uncertainties [33] were also determined according to EN ISO/IEC 17025:2005. Type A and Type B uncertainties were first calculated and used to determine the combined standard uncertainty (*u*_c_) and the expanded uncertainty (*U*). To prove the applicability of the proposed method in the analysis of volatile compounds, the percentage relative recovery (%RR), the trueness, and the matrix effect (ME) were calculated. The analytes that were not present in the investigated samples were added at different concentrations, namely 10.0, 30.0, and 50.0 mg kg^−1^.

Percentage relative recovery was calculated as:

1 $$RR\;(\%) = \frac{{C}_{\text{m}} }{{C}_{\text{add}}}\times 100$$

where *C*_m_ is the concentration of the analyte measured after fortification of the sample and *C*_add_ is the level of fortification of the analyzed sample.

Based on the method described in ISO 5725–4;1994 [34], the trueness was calculated using the following equation:

2 $$\text{Trueness }=1- \frac{\text{mean recovery corrected concentration detected }}{\text{fortified level}}$$

where the “mean recovery corrected concentration detected” is calculated by correcting the experimental determined concentration with the relative recovery (Eq. 4), while the “fortified level” is the expected concentration after spiking. In line with the requirements of Regulation (EU) 2021/808 [31], the minimum range of trueness should be − 0.20 to + 0.20 for the samples under investigation, as the mass of the target substances is higher than 10 µg kg^−1^.

The matrix effect was calculated by generating two calibration curves with the same concentration range (1.0 to 50.0 mg kg^−1^). The first one was built-up by diluting the standard mixture in ethanol (blank) and submitting the sample to the PT-µSPE, and the second one by spiking the perfumes with the same standard mixture before PT-µSPE. The matrix effect was calculated as the slope of the calibration curve with the sample matrix (*k*_1_) compared to the slope of the curve without sample (*k*) using the following equation [35]:

3 $$\text{ME }= 1- \frac{{k}_{1}}{k} \times 100$$

Moreover, the recovery of the analytes originally present in the samples was assessed by performing successive elutions on the same pipette-tip device previously loaded with the perfumes and calculating the percentage of each compound recovered during the first extraction.

According to IUPAC [36], relative error was calculated as follows:

4 $$\text{Relative error }= \frac{\text{accepted value }-\text{obtained value}}{\text{accepted value}}$$

and was considered acceptable in the range ± 0.20 [14].

### Overall evaluation of the method

AGREE [21], BAGI (Blue Applicability Grade Index) [37], NQS (Need, Quality, Sustainability) [26], WAC (White Analytical Chemistry) [24], and RGB (Red, Green, Blue) [23] metric tools were used to evaluate the greenness and overall performance of the developed methods.

The reference SPE and pipette-tip micro-solid-phase extraction methods, as well as the direct injection method, were evaluated. The reference extraction process (see section “Extraction methods”) was followed by separate GC-FID (for semi-quantitative purposes) and GC–MS analysis (for identification and quantitation purposes).

### Data elaboration

The software used for acquisition and processing of the data collected with MS was GCMSsolution^®^ 4.30, while the FID data were acquired and processed with GCsolution^®^ software (Shimadzu, Milan, Italy). Excel (Microsoft Office, v.2016) was used for the method validation and for the calculation of the RGB (based on the tool developed by Nowak et al. [23]), WAC [24], and NQS [26] indices. The Analytical GREENess Calculator (AGREE v0.4 2020) [21] was used to calculate the greenness score of the methods. The BAGI Web Calculator [37] was used to calculate the productivity of the methods. GraphPad Prism 10.2.1 (GraphPad Software, LLC, USA) was used to process the data and create the graphics.

## Results and discussion

### Development of simultaneous quantitative and semi-quantitative chromatographic method

In the present study, the first step was to develop a chromatographic method with simultaneous dual detection (MS and FID). FID detection is applied to determine the relative percentage composition (semi-quantification) of the volatile components in the Perfumes, while MS is adopted for the accurate quantification of the target analytes (see section “Analysis conditions”). In particular, a very simple passive Y-shaped glass splitter was used to connect the main column to the two detectors via deactivated capillary column segments. A 20 m long × 0.18 mm *d*_*c*_ column coated with an apolar stationary phase was used for the separation. This column geometry enables to shorten the analysis time (compared to columns with a larger inner diameter) without special instrumental requirements [38]. At the same time, the column was coated with a thicker film of stationary phase (0.50 µm), not only to prevent damages because of water (when the Perfumes are directly injected), but also to improve its loadability, and thereby the sensitivity of the method, since the amount of analyte reaching each detector is considerably lower because of dual-detection splitting. Before installing the capillaries, the column geometry, flow rate, and pressure balance between the two detectors were optimized. For this purpose, the Effluent Splitter Pressure Calculator from Agilent was used (see Figure S1a). Different column geometries (lengths and inner diameters) were tested to achieve a sufficient pressure at the end of the main column and a suitable flow rate for both capillaries. The optimal conditions resulted to be a 2 m × 0.18 mm *d*_*c*_ capillary connected to the FID detector and a 1 m × 0.1 mm *d*_*c*_ segment connected to the MS. Under these conditions, a splitter pressure of 15.97 kPa and a percent flow to the FID detector of 57.13%, ensuring good sensitivity for both quantitative and semi-quantitative evaluations, were obtained. The analyses were performed in constant pressure mode to avoid changes in the percentage flow ratio during the temperature run. Figure S1b-d compare the reference GC–MS pattern of Perfume 5 obtained with single detection (Fig. S1b) and the GC–MS (Figure S1c) and GC-FID (Figure S1d) profiles obtained with the optimized GC–MS/FID approach.

**Fig. 2:**
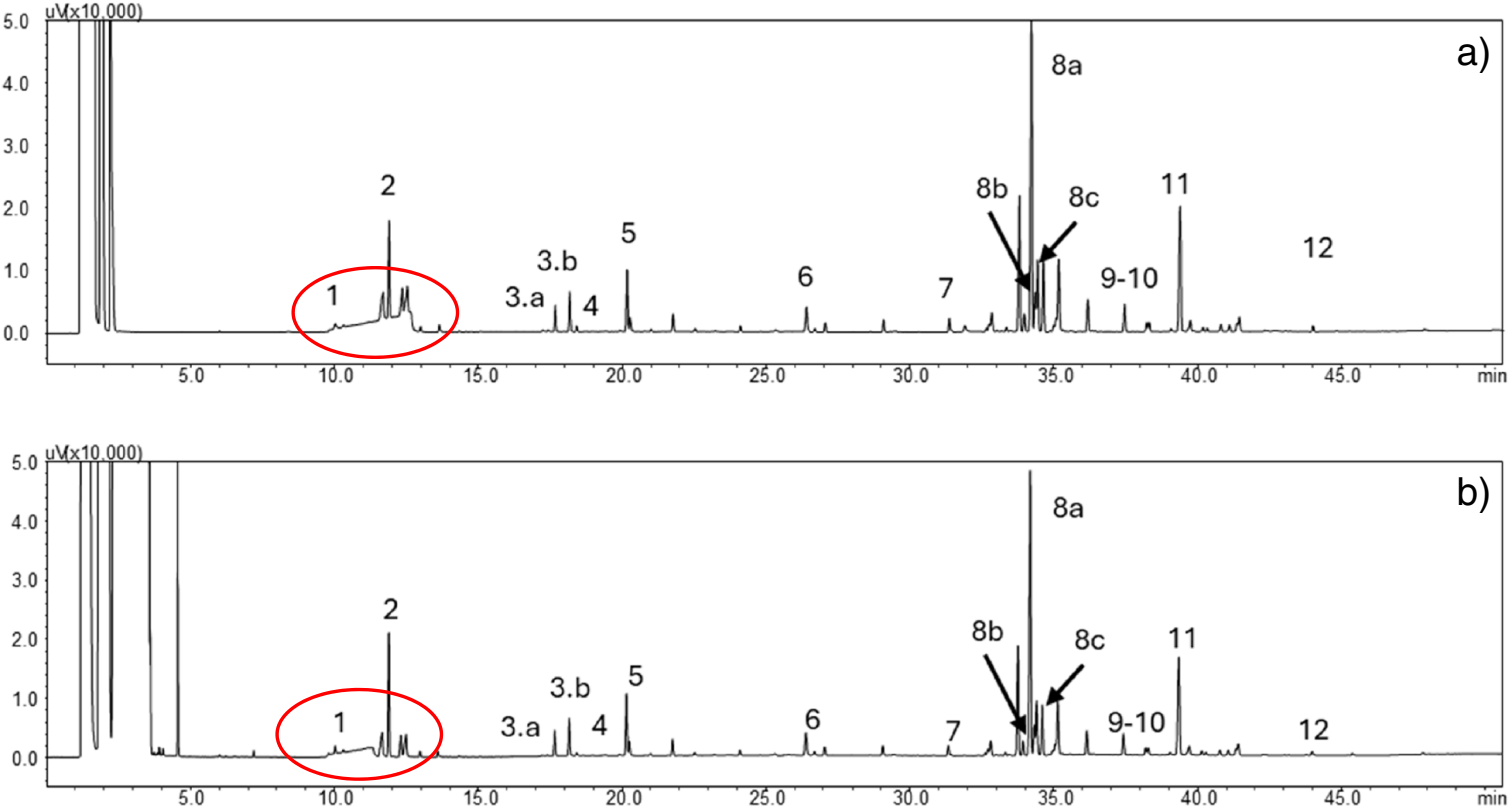
Chromatographic profile of the analyzed samples: Perfume 5 (**a**) directly injected and (**b**) after extraction with PT-µSPE. Legend: 1, β-pinene; 2, limonene; 3a, ethyl linalool 1; 3b, ethyl linalool 2; 4, 1,4-dibromobenzene; 5, linalyl acetate; 6, *trans*-β-caryophyllene; 7, helional; 8a, iso e super 1; 8b, iso e super 2; 8c, iso e super 3; 9, benzyl benzoate; 10, ambroxide; 11, galaxolide; 12, 4,4′-dibromobiphenyl. The red circle indicates the zone of elution of glycerin

The investigated Perfumes were first directly injected for GC–MS/FID analysis after a 50-fold dilution in ethanol to avoid the introduction in the system of a large amount of water and cosmetic agents. This simple procedure does not require sample preparation, and it is not time-consuming nor expensive. However, despite its simplicity and speed, it suffers the co-injection of water and cosmetic agents in significant quantities beside the volatile analytes of interest. The cosmetic agents tend to interfere with the chromatographic profile because they coelute with some of the target analytes. Moreover, because of their medium to low volatility, contamination of the liner or column head may occur, resulting in lower repeatability and accuracy of the data after a few injections and a short column lifetime. The pattern of Perfume 5, analyzed by direct injection, is shown in Fig. 2a, while those of Perfume 1 in Figure S2a. The results show the interference of cosmetic additives (in particular glycerin) in the chromatographic profile, as it co-elutes with several peaks of interest, making their quantification rather difficult. Table 1 reports the results of semi-quantification (normalized percentage composition) of the tested samples injected directly after dilution in ethanol.

**Table 1:** Relative percentage composition of target compounds in the investigated Perfumes directly injected (*n* = 3)

Compounds	Relative composition (%) ± standard deviation			
	**Perfume 1 DI**	**Perfume 2 DI**	**Perfume 3 DI**	**Perfume 4 DI**
Benzaldehyde	1.1 ± 0.01	1.0 ± 0.02	1.0 ± 0.03	0.9 ± 0.05
Coumarin	5.8 ± 0.03	5.6 ± 0.01	6.6 ± 0.01	6.4 ± 0.01
Iso e super 1	10.8 ± 0.01	10.5 ± 0.00	10.1 ± 0.01	10.2 ± 0.00
Iso e super 2	1.0 ± 0.03	1.1 ± 0.01	1.3 ± 0.01	1.3 ± 0.01
Iso e super 3	2.0 ± 0.01	2.0 ± 0.01	2.2 ± 0.00	2.2 ± 0.01
Ambroxide	1.3 ± 0.01	1.4 ± 0.00	1.6 ± 0.02	1.6 ± 0.01
Benzyl benzoate				
Habanolide 1	4.6 ± 0.02	4.6 ± 0.00	4.9 ± 0.00	4.9 ± 0.00
Habanolide 2	3.0 ± 0.01	3.1 ± 0.01	3.4 ± 0.01	3.3 ± 0.01
Habanolide 3	2.9 ± 0.02	3.0 ± 0.01	3.3 ± 0.03	3.2 ± 0.00
Ambrettolide	1.2 ± 0.01	1.3 ± 0.01	1.5 ± 0.01	1.4 ± 0.01
	**Perfume 5 DI**	**Perfume 6 DI**	**Perfume 7 DI**	**Perfume 8 DI**
Limonene	9.2 ± 0.01	8.3 ± 0.00	3.6 ± 0.02	5.1 ± 0.02
Ethyl linalool 1	1.6 ± 0.02	1.6 ± 0.03	1.4 ± 0.05	1.4 ± 0.04
Ethyl linalool 2	2.5 ± 0.01	2.5 ± 0.02	2.2 ± 0.02	2.1 ± 0.01
Iso e super 1	22.7 ± 0.01	20.8 ± 0.02	22.5 ± 0.01	22.4 ± 0.01
Iso e super 2	2.2 ± 0.03	2.3 ± 0.02	2.7 ± 0.02	2.5 ± 0.00
Iso e super 3	4.1 ± 0.02	4.1 ± 0.05	4.7 ± 0.01	4.5 ± 0.01
Ambroxide	1.7 ± 0.00	1.9 ± 0.02	2.2 ± 0.03	2.1 ± 0.01
Benzyl benzoate				
Galaxolide	10.2 ± 0.00	9.8 ± 0.01	11.4 ± 0.03	11.1 ± 0.00
Linalyl acetate	4.1 ± 0.00	3.9 ± 0.00	3.3 ± 0.02	3.2 ± 0.01
* Trans-β-*Caryophyllene	1.9 ± 0.01	1.4 ± 0.02	1.8 ± 0.06	2.4 ± 0.41
Helional	0.7 ± 0.00	0.7 ± 0.03	1.0 ± 0.01	0.9 ± 0.09

### Optimization of the sample preparation method

Based on an extraction method developed for the isolation of the volatile fraction of wine [29], a solid-phase extraction method was tested (“reference method") and then optimized, using Perfume 1 as a model sample. The optimization aimed to minimize the amount of material used and to simplify the procedure (by reducing the number of individual steps and thus the extraction time), while achieving accurate results not only in terms of target analyte quantification but also on the fingerprint of the volatile components (evaluated as relative percentage composition). The extraction efficiency of the tested methods was therefore evaluated by comparing the percentage composition of some representative analytes of different polarity and volatility and belonging to different chemical classes (benzaldehyde, coumarin, iso e super, ambroxide, benzyl benzoate, habanolide, ambrettolide) with the composition obtained by direct injection of the Perfumes. The results, expressed as relative error, obtained for each extraction method tested are shown in Table 2, while the detailed extraction conditions are reported in Table S3.

**Table 2:** Average relative errors of the relative percentage composition of the main components of Perfume 1 submitted to different extraction processes. For the extraction conditions, see Table S3

Compounds	Reference	Test 1	Test 2	Test 3	Test 4	Test 5	Test 6	Test 7	Test 8	Test 9	PT-µSPE
Benzaldehyde	0.10	0.09	0.13	0.11	− 0.07	− 0.14	− 0.05	− 0.31	− 0.14	− 0.43	0.01
Coumarin	0.35	0.36	0.4	0.41	0.22	0.26	− 0.22	− 0.6	− 0.10	− 0.55	0.01
Iso e super 1	− 0.55	− 0.52	− 0.55	− 0.53	− 0.37	− 0.39	− 0.03	0.64	− 0.15	0.59	− 0.06
Iso e super 2	− 0.41	− 0.38	− 0.40	− 0.41	− 0.27	− 0.32	− 0.03	0.63	− 0.15	0.60	− 0.13
Iso e super 3	− 0.42	− 0.40	− 0.42	− 0.40	− 0.31	− 0.35	0.00	0.60	− 0.12	0.56	− 0.20
Benzyl benzoate/ambroxide	− 0.36	− 0.32	− 0.35	− 0.30	− 0.26	− 0.30	− 0.01	0.60	− 0.14	0.58	− 0.18
Habanolide 1	− 0.51	− 0.47	− 0.52	− 0.45	− 0.33	− 0.36	− 0.04	0.70	− 0.15	0.67	− 0.12
Habanolide 2	− 0.48	− 0.43	− 0.48	− 0.48	− 0.30	− 0.34	− 0.02	0.70	− 0.15	0.66	− 0.19
Habanolide 3	− 0.47	− 0.42	− 0.47	− 0.40	− 0.30	− 0.34	− 0.02	0.68	− 0.14	0.65	− 0.19
Ambrettolide	− 0.41	− 0.34	− 0.40	− 0.39	− 0.28	− 0.32	− 0.04	0.74	− 0.16	0.70	− 0.12

In the reference method (Fig. 1a), 4.5 g of sample is loaded onto 2.5 g of sorbent material (EXtrelut^®^ NT) and the target analytes are recovered with 12.5 g of elution solvent (cyclohexane). The method was then optimized in terms of amount of sample, sorbent, and solvent. The amount of sample was decreased to 2.5 g thus establishing a ratio of 1:1 between the sample and the sorbent, and the solvent was lowered to 7 g (method defined as “test 1”). The results (Table 2) did not show significant differences when compared to the percentage areas of the reference method. The quantity of both materials was then reduced by a factor of 2.5 (method “test 2”), i.e., with 1 g of sample, 1 g of sorbent, and 2.5 g of solvent accurately weighed. Again, no significant differences with the previous method were observed.

A further step of the optimization concerned the selection of the adsorbent material. EXtrelut^®^ NT is a patented, inert diatomaceous earth with a large pore volume. A naturally occurring and untreated siliceous soft sedimentary rock (Celite 545^®^) was tested also to reduce extraction costs (by 6 folds) (“test 3”). The resulting GC patterns with the two sorbent phases were fully superimposable and celite was therefore selected for the subsequent extractions. Last, the method was miniaturized by tenfold decreasing the amounts of sample and sorbent and reducing five-times the amount of the extraction solvent (method “test 4”), showing results comparable to those obtained with the previous method (see Table 2). Therefore, the method “test 4” was chosen for further optimization.

Due to the complexity of a fragrance concentrate containing apolar and more polar compounds, a further important point in the optimization of the extraction step is the choice of the eluent, which should allow to extract as many analytes as possible without releasing water and cosmetic additives, at the same time ensuring optimal compatibility with the GC injection and not affecting the chromatographic pattern. Therefore, different combinations of solvents were tested (see Table S3). Cyclohexane (LogP = 3.4) showed the worst results in terms of relative errors (with values in the range of − 0.37 and 0.22), due to its apolarity that enables a better extraction of apolar compounds at the detriment of polar compounds. Cyclohexane was replaced by heptane (LogP = 4.4), considered by some authors as less toxic to the environment and human health [39] (“test 5”). Similar results were obtained with relative error values ranging from − 0.39 to 0.26. Therefore, a more polar solvent was added to the elution mixture. Ethyl acetate (LogP = 0.7), a bio-based solvent, was first tested because it is more polar than cyclohexane and heptane and it also shows lower toxicity to the environment and human health, as stated by the Solvent Selection Guidelines [32–34]. A mixture of heptane and ethyl acetate in a 50:50 ratio (“test 6”) produced an improvement in extraction efficiency of all fragrance components with relative error values ranging from − 0.05 to 0.00 for all target compounds, with the only exception of coumarin which shown a value of − 0.22. A further experiment was carried out by replacing ethyl acetate with acetonitrile, in a 50:50 ratio (“test 7”). Acetonitrile (LogP = − 0.3) was tested because it is more polar than ethyl acetate. The results were similar in terms of relative errors for both polar and apolar compounds, but it extracted a higher amount of cosmetic agents, in particular glycerin (Figure S3). The combinations heptane/ethyl acetate and heptane/acetonitrile were then tested at a ratio of 70:30 (“test 8” and “test 9”, respectively) to limit the extraction of interferences and improving the extraction of the compounds of interest. The “test 8”, resulted in a similar trend of errors for both polar and apolar compounds, but in the co-extraction of a lower amount of cosmetic agents, while for “test 9” the presence of interferences was still significant. The mixture adopted for “text 8” was therefore considered optimal.

The last step concerned the simplification of the extraction process with a further reduction of the amount of sorbent, solvent and sample used and of the number of steps required, also in view of a possible automation of the process. A PT-µSPE was therefore developed. PT-µSPE did not required either centrifugation or vortex and spatula mixing because sample, sorbent, and solvent are easily dispersed during aspiration. Indeed, the process was streamlined by simple aspiration and dispensing, which reduced the number of steps and shortened the overall extraction time (see section “Extraction methods”). The optimized procedure (Fig. 1b) reduced the amount of sample and sorbent a factor of 10, so that only 10 mg of diluted sample and celite are needed, while the extraction solvent was reduced by a factor of 2.5 to obtain a final volume compatible with direct analysis by GC–MS/FID. The PT-µSPE approach requires only aspiration/dispensing cycles which greatly simplifies the procedures and reduces sample preparation time. Figure 2b and Figure S2b show the chromatographic patterns of the analyzed samples after extraction with the developed PT-µSPE method for Perfumes 5 and 1, respectively. The results (see Table 2) show that the PT-µSPE method provides values of relative error within ± 0.20 for all target analytes and therefore a representative fingerprint of the fragrance concentrate in the final water-based perfume.

### PT-µSPE validation

The developed PT-µSPE method was validated on Perfumes 5 and 6 to evaluate the reliability of the developed approach for samples containing different cosmetic agents. The validation was performed in accordance with Regulation (EU) 2021/808 [31] and the following parameters were calculated: linearity, relative recovery, trueness, repeatability, reproducibility, and matrix effect. The results are summarized in Table 3 and Table S4.

**Table 3:** Analytical figure of merits of the quantitative PT-µSPE GC–MS/FID method for the investigated analytes in Perfumes 5 and 6. Combined Standard Uncertainty (*u*_*c*_) and Expanded Uncertainty (*U*) were calculated at 10 mg kg^−1^ level

	**Repeatability (RSD%) (*n* = 9)**	**Reproducibility (RSD%) (*n* = 27)**	**Relative recovery (RR%)**	**Trueness**	**Matrix effect**	***u***_**c**_	***U***
PT-µSPE Perfume 5							
Benzaldehyde	0.9%	2.4%	94%	− 0.18	12%	0.48	0.96
Coumarin	0.1%	0.9%	91%	0.01	4%	0.56	1.1
Habanolide 1	0.6%	0.7%	121%	− 0.20	4%	0.55	1.1
Habanolide 2	0.6%	2.0%	111%	0.07	3%	0.20	0.39
Habanolide 3	1.3%	1.2%	87%	0.01	13%	0.15	0.29
Ambrettolide	1.5%	1.4%	118%	0.06	4%	0.75	1.5
PT-µSPE Perfume 6							
Benzaldehyde	1.7%	2.9%	96%	0.08	19%	0.48	0.96
Coumarin	0.1%	3.0%	77%	0.17	4%	0.56	1.1
Habanolide 1	0.5%	3.9%	114%	0.13	12%	0.40	0.80
Habanolide 2	1.9%	3.9%	86%	0.18	11%	0.20	0.39
Habanolide 3	0.4%	4.8%	76%	0.13	19%	0.12	0.24
Ambrettolide	1.7%	2.1%	99%	0.23	12%	0.62	1.3

Quantification was based on the calibration curves obtained by analyzing standard mixtures in two concentration ranges (from 0.2 to 50 mg kg^−1^ and from 100 to 500 mg kg^−1^). The linearity of the response was evaluated by calculating the coefficient of determination (*R*^2^) of the calibration curves subjected to the extraction process, with the peak areas of the target analytes normalized to those of the internal standard 1,4-dibromobenzene (added at a concentration of 50 mg kg^−1^). The coefficients of determination ranged from *R*^2^ = 0.988 to *R*^2^ = 0.999, showing good analytical linearity for the proposed method (Table S4).

The limit of detection (LOD) and limit of quantification (LOQ) were experimentally determined as the concentration of the standard mixture solutions extracted by the proposed method, which give peaks with 3 and 10 times the signal-to-noise ratio of the standard mixture solutions extracted by the proposed method, respectively. The LOD values ranged from 0.01 to 0.50 mg kg^−1^, while LOQ values ranged from 0.07 to 3.30 mg kg^−1^ (Table S4).

Extraction efficiency and accuracy were evaluated by percentage relative recovery (%RR) and trueness by spiking the samples with the two standard mixtures at three validation levels (namely, 10, 30, 50 mg kg^−1^) in three independent extractions analyzed in triplicate. The results, calculated for the compounds not originally present in the investigated Perfumes, are reported in Table 3. The relative recovery, calculated at two concentration levels (namely, 10 and 50 mg kg^−1^), ranged from 87 to 121% for Perfume 5 and from 76 to 114% for Perfume 6, indicating a good extraction efficiency of the proposed method. The trueness was calculated for the same compounds at a concentration of 10 mg kg^−1^. The results obtained were between − 0.20 and + 0.07 and between 0.08 and 0.23 for Perfumes 5 and 6, respectively, which is in line with the requirements of Regulation (EU) 2021/808 [31] (see section “Validation of the method”), with the only exception of ambrettolide (0.23). It is important to note that both relative recovery and trueness are usually considered a measure of a method’s accuracy. However, relative recovery provides an indication of the extraction efficiency and trueness is calculated by correcting the mean analyte concentration using the recovery, meaning these two values are not directly correlated. Correcting the obtained concentration with relative recovery can often lead to highly accurate results, as seen with habanolide 3 in perfume 5 (%RR, 87%; trueness, 0.01). In some cases, however, the opposite occurs, as it is for ambrettolide in Perfume 6, where despite a high relative recovery (%RR, 99%), trueness is 0.23. The recovery of the analytes originally present in the Perfume 5 and Perfume 6 was also assessed by performing successive elutions on the same pipette-tip device previously loaded with the perfumes and calculating the percentage of each compound recovered during the first extraction. The results showed a complete recovery of the analytes after two extractions, with recoveries exceeding 90% in the first elution for both samples. Specifically, the recoveries ranged from 90.5 to 94.5% for Perfume 5 and from 90.8 to 93.6% for Perfume 6.

The precision of the method was assessed by measurement uncertainties, repeatability, and intra-laboratory reproducibility. The results of the combined standard uncertainty (*u*_c_) and expanded uncertainty (*U*) for the lowest concentration (10 mg kg^−1^) are reported in Table 3. The results were satisfactory, with expanded uncertainty for all compounds below 15%. Repeatability showed an RSD% between 0.1 and 1.5% (*n* = 9) for Perfume 5, while for Perfume 6, the RSD% was between 0.1 and 1.9%. The intra-laboratory reproducibility (*n* = 27) was between 0.7 and 2.4% for Perfume 5, and between 2.1 and 4.8% for Perfume 6. The matrix effect (ME) was again determined for the Perfumes 5 and 6 by comparing the slope of the curves obtained spiking the Perfumes and the curve without Perfumes. Table 3 shows the ME value, which ranged from 3 to 13% for Perfume 5, and from 4 to 19% for Perfume 6. These results show that the matrix effect has no statistically significant influence on the extraction process [42, 43].

### Analysis of commercial samples by PT-µSPE and direct injection

The validated PT-µSPE method was then applied to eight commercial perfumes for quantitative and semi-quantitative characterization. Table 4 reports the concentrations of the target analytes in the finished products obtained by direct injection and after extraction. Quantification of the directly injected samples was carried out utilizing specific calibration curves and the results were compared to those obtained by the validated PT-µSPE method, taken as a reference. The quantification of the target analytes in the Perfumes 1, 2, 3, and 4 showed values of relative error significantly different from an acceptable range (± 0.20) for most analytes, with the only exception of benzaldehyde in Perfumes 2 and 3 (− 0.13 and − 0.15, respectively), coumarin in Perfumes 3 and 4 (− 0.03 and − 0.16), and benzyl benzoate (0.00 and − 0.20). Also the relative error results for Perfumes 5, 6, 7, and 8 exceed ± 0.20 for several analytes. This deviation is quite high for all analytes for Perfumes 7 and 8, while for Perfumes 5 and 6 only some compounds are significantly out of range, such as for limonene, iso e super isomer 1, ambroxide, benzyl benzoate, helional, and galaxolide. Moreover, higher values for reproducibility (*n* = 27), i.e., up to 8.8%, were obtained compared to PT-µSPE (up to 5.5%). The same trend can be observed in terms of combined standard uncertainty and expanded uncertainty (see Table S6). These results confirmed that direct injection of cosmetic additives can interfere with accurate quantification of water-based perfumes and underline the need for pretreatment of samples prior to analysis.

**Table 4:** Concentration (g kg^−1^) of target compounds in the perfumes obtained by PT-µSPE (*n* = 9) and directly injected (*n* = 3). Relative errors are reported in brackets

Compounds	Concentration (g kg^−1^) ± expanded uncertainty				Concentration (g kg^−1^) ± expanded uncertainty (*relative errors*)			
	**PT-µSPE Perfume 1**	**PT-µSPE Perfume 2**	**PT-µSPE Perfume 3**	**PT-µSPE Perfume 4**	**DI Perfume 1**	**DI Perfume 2**	**DI Perfume 3**	**DI Perfume 4**
**Benzaldehyde**	34.45 ± 0.32	36.97 ± 0.37	0.595 ± 0.012	1.233 ± 0.017	27.2 ± 3.1 (*0.21*)	32.5 ± 0.00 (− *0.13*)	0.68 ± 0.09 (− *0.15*)	1.39 ± 0.00 (− *0.57*)
**Coumarin**	49.7 ± 4.8	52.9 ± 6.4	2.07 ± 0.24	2.30 ± 0.27	71.9 ± 8.8 (*0.31*)	79.7 ± 0.02 (*0.33*)	2.13 ± 0.01 (− *0.03*)	3.85 ± 0.02 (− *0.16*)
**Iso e super 1**	71.4 ± 6.9	67.3 ± 8.3	2.42 ± 0.28	3.02 ± 0.35	91.3 ± 10 (*0.22*)	97.3 ± 0.01 (*0.30*)	3.01 ± 0.01 (− *0.24*)	5.63 ± 0.01 (− *0.38*)
**Iso e super 2**	7.87 ± 0.76	7.77 ± 0.90	0.345 ± 0.040	0.346 ± 0.040	10.8 ± 1.2 (*0.26*)	13.9 ± 0.36 (*0.43*)	0.39 ± 0.02 (− *0.13*)	0.76 ± 0.02 (− *0.30*)
**Iso e super 3**	13.6 ± 1.3	13.1 ± 1.5	0.537 ± 0.062	0.595 ± 0.069	17.5 ± 2.0 (*0.22*)	18.9 ± 0.01 (*0.31*)	0.65 ± 0.01 (− *0.22*)	1.30 ± 0.02 (− *0.38*)
**Ambroxide**	7.84 ± 0.75	6.78 ± 0.79	0.605 ± 0.070	0.335 ± 0.039	17.9 ± 2.1 (*0.56*)	19.1 ± 0.02 (*0.64*)	0.78 ± 0.02 (− *0.29*)	1.62 ± 0.02 (− *0.58*)
**Benzyl benzoate**	3.30 ± 0.32	3.17 ± 0.37	0.100 ± 0.012	0.151 ± 0.017	5.05 ± 0.58 (*0.34*)	5.4 ± 0.01 (*0.40*)	0.10 ± 0.01 (*0.00*)	0.21 ± 0.02 (− *0.20*)
**Habanolide 1**	41.9 ± 4.0	40.6 ± 4.7	0.87 ± 0.10	1.84 ± 0.21	65.9 ± 9.1 (*0.36*)	71.7 ± 0.00 (*0.4*3)	1.64 ± 0.02 (− *0.89*)	3.03 ± 0.02 (− *1.13*)
**Habanolide 2**	27.9 ± 2.7	27.7 ± 3.2	0.608 ± 0.070	1.24 ± 0.14	44.3 ± 5.2 (*0.37*)	48.1 ± 0.00 (*0.42*)	1.16 ± 0.00 (− *0.91*)	2.13 ± 0.01 (− *1.15*)
**Habanolide 3**	26.4 ± 2.5	26.4 ± 3.1	0.586 ± 0.068	1.18 ± 0.14	42.8 ± 6.9 (*0.38*)	46.7 ± 0.00 (*0.43*)	1.11 ± 0.02 (− *0.90*)	2.03 ± 0.00 (− *1.12*)
**Ambrettolide**	10.7 ± 1.0	10.5 ± 1.2	0.348 ± 0.040	0.491 ± 0.057	18.0 ± 2.1 (*0.40*)	19.2 ± 0.01 (*0.45*)	0.46 ± 0.02 (− *0.31*)	0.86 ± 0.02 (− *0.68*)
	**PT-µSPE** **Perfume 5**	**PT-µSPE** **Perfume 6**	**PT-µSPE** **Perfume 7**	**PT-µSPE Perfume 8**	**DI Perfume 5**	**DI Perfume 6**	**DI Perfume 7**	**DI Perfume 8**
**Limonene**	69.0 ± 9.0	56.1 ± 7.4	0.825 ± 0.095	2.085 ± 0.095	36.1 ± 0.01 (*0.48*)	45.7 ± 0.02 (*0.19*)	1.05 ± 0.01 (− *0.27*)	2.50 ± 0.00 (− *0.20*)
**Ethyl linalool 1**	15.6 ± 1.8	15.0 ± 1.7	0.440 ± 0.051	0.685 ± 0.051	16.0 ± 0.01 (− *0.03*)	16.9 ± 0.02 (− *0.11*)	0.60 ± 0.01 (− *0.37*)	0.95 ± 0.01 (− *0.39*)
**Ethyl linalool 2**	22.7 ± 2.6	22.9 ± 2.6	0.665 ± 0.077	1.034 ± 0.077	23.8 ± 0.01 (− *0.05*)	25.8 ± 0.02 (− *0.11*)	0.96 ± 0.02 (− *0.45*)	1.51 ± 0.01 (− *0.46*)
**Iso e super 1**	87 ± 12	128 ± 16	5.82 ± 0.68	8.57 ± 0.99	145.5 ± 0.02 (− *0.40*)	185.2 ± 0.01 (− *0.30*)	7.78 ± 0.00 (*0.34*)	6.07 ± 0.01 (*0.29*)
**Iso e super 2**	18.7 ± 2.2	20.4 ± 2.5	0.87 ± 0.10	1.32 ± 0.15	22.1 ± 0.03 (− *0.15*)	22.5 ± 0.00 (− *0.10*)	1.08 ± 0.01 (− *0.24*)	1.72 ± 0.00 (− *0.30*)
**Iso e super 3**	32.9 ± 4.0	32.9 ± 3.9	1.39 ± 0.16	2.11 ± 0.24	30.9 ± 0.03 (− *0.06*)	37.3 ± 0.01 (− *0.12*)	1.91 ± 0.01 (− *0.38*)	2.88 ± 0.01 (− *0.37*)
**Ambroxide**	10.7 ± 1.2	12.3 ± 1.4	0.690 ± 0.080	1.04 ± 0.12	16.6 ± 0.03 (− *0.35*)	18.5 ± 0.02 (− *0.33*)	0.98 ± 0.02 (− *0.42*)	1.65 ± 0.02 (− *0.58*)
**Benzyl benzoate**	6.95 ± 0.80	7.56 ± 0.90	0.253 ± 0.029	0.343 ± 0.040	11.8 ± 0.03 (− *0.40*)	12.7 ± 0.02 (− *0.40*)	0.37 ± 0.01 (− *0.45*)	0.57 ± 0.02 (− *0.67*)
**Galaxolide**	56.8 ± 9.1	93 ± 12	4.62 ± 0.54	6.89 ± 0.80	106.2 ± 0.02 (*0.46*)	129.3 ± 0.01 (*0.28*)	6.3 ± 0.00 (− *0.36*)	9.4 ± 0.02 (− *0.37*)
**Linalyl acetate**	27.2 ± 3.1	31.6 ± 3.4	0.529 ± 0.061	0.821 ± 0.095	27.1 ± 0.01 (*0.00*)	30.3 ± 0.02 (− *0.04*)	0.86 ± 0.01 (− *0.63*)	1.35 ± 0.01 (− *0.64*)
***trans*-β-Caryophyllene**	16.1 ± 1.9	18.6 ± 5.5	0.352 ± 0.041	0.542 ± 0.063	17.6 ± 0.01 (− *0.09*)	17.9 ± 0.01 (− *0.04*)	0.55 ± 0.01 (− *0.56*)	0.86 ± 0.00 (− *0.59*)
**Helional**	8.42 ± 0.97	9.4 ± 1.2	0.291 ± 0.034	0.393 ± 0.045	16.5 ± 0.03 (− *0.49*)	16.3 ± 0.03 (*0.42*)	0.46 ± 0.00 (− *0.59*)	0.72 ± 0.00 (− *0.83*)

To verify the reliability of the fingerprint information after PT-µSPE, the relative percentage composition of the fragrance obtained with this method was compared to that obtained by direct injection. The results are reported in Table S5. For Perfumes 1–4 (characterized by a similar composition), the relative errors were between − 0.37 and 0.23, while for Perfumes 5–8 they were between − 0.27 and 0.30, with the only exception of limonene in Perfume 7 whose error was 0.54, indicating that in most cases, the proposed method has no effect on the percent composition, allowing the extraction of all target analytes of the perfumes.

### Assessment of the method performance

As reported in the “Introduction”, several metrics have been developed in recent years to calculate the performance of analytical methods [44]. According to the White Analytical Chemistry (WAC) [24] approach, the aim of this work was to estimate the overall performance of the developed PT-µSPE method, in terms of analytical performance, greenness, and practical efficiency, that is a tool that laboratories can easily apply to compare the performance of their methods. Indeed, WAC was developed to balance all aspects of an analytical method in view of industrial quality controls. In this study, sample preparation and chromatographic separation were the most important and critical elements of the analytical workflow to be evaluated. Sample preparation often requires the use of different (and potentially toxic) materials and reagents, which can be consumed in large quantities, as well as the use of energy-intensive instruments and can be time-consuming. On the other hand, gas chromatographic analyses require high consumption of energy, time, and (non-renewable) gas for each analysis [45]. The developed PT-µSPE method in combination with GC–MS/FID allowed to improve several of these aspects as well as to obtain reliable information about the sample. The performance of the optimized method was therefore compared to the “reference method” (see section “Extraction methods”) using a not miniaturized sample preparation method [29] and separate analyses for quantification and semi-quantification and with GC–MS/FID analysis by direct injection (“direct injection” method).

AGREE [18] was first applied to have an idea of the environmental performance of the methods, since it is one very often used to evaluate the compliance of an analytical method with the 12 principles of Green Analytical Chemistry (GAC). However, it should be emphasized that it does not allow to determine the environmental impact of a method. Figure S4 shows the comparison of AGREE results for the (a) the reference method, (b) the direct injection, and (c) the optimized PT-µSPE method. All 12 principles are considered with the same weighting. Some criticisms of the reference method were emphasized, as its final score is 0.49. The low score of the reference method is mainly related to the energy consumption (1.23 kWh measured with a power meter plug), as a duplicate analysis must be performed for each sample and uses a considerable amount (12.5 g) of a toxic solvent (cyclohexane, a non-renewable organic solvent toxic to the environment and human health). Other criticisms concern principles 10 and 11, which refer to the renewability and toxicity of the reagents and received a score of 0.0 and 0.15, respectively, due to the use of cyclohexane, a non-renewable organic solvent that is toxic to the environment and human health. The result also highlights that a reduction in the amount of materials used and a miniaturization of the process (Principle 5) are desirable, because it would also lead to less waste production (Principle 7). The AGREE score drastically increases for the direct injection method (score 0.84) as no sample preparation is required and only 50 mg of the sample was used as well as a renewable and non-toxic reagent (ethanol for dilution). Finally, the developed PT-µSPE method has a final score (0.79), that is lower than the direct injection method due to the use of materials and solvents (i.e., heptane) that are non-renewable. On the other hand, although sample preparation is required, a lower number of steps (two steps) is needed compared to the reference methods (five steps), which reduces the impact on the final score. In both cases, the energy consumption was significantly lower (0.79 kWh), as the dual GC–MS/FID setup allows performing only one analysis for a complete characterization of the sample.

To assess the greenness of an analytical method, it is also possible to calculate greenhouse gas (GHG) emissions, expressed in terms of carbon dioxide equivalents (kgCO_2_eq). Although these calculations do not constitute a full life cycle assessment (LCA), because the system boundaries have not been defined and several upstream and downstream impacts have not been considered [22], they enable to evaluate the trend of carbon footprint of the investigated methods considering the same elements evaluated with the other metric tools. The results (Figure S5) provide a comparison of the GHG emissions associated with the three analytical methods, highlighting significant differences in their environmental impacts. The reference method, with a total emission of 0.64 kgCO_2_eq, exhibits the highest environmental footprint, primarily due to its higher reliance on electricity and greater consumption of solvents and consumables. In contrast, direct injection and PT-µSPE demonstrate considerably lower emissions, at 0.35 kgCO_2_eq because require only trace amounts of solvents. Both these methods are therefore more environmentally sustainable compared to the reference method, but the choice between them might depend on other factors such as cost, efficiency, or analytical performance.

The classification by the Blue Applicability Grade Index (BAGI) focuses instead on the productivity of the method. This metric tool was applied to the method investigated in this study and, as shown in Figure S6, no significant differences were found for the methods requiring sample preparation (final score around 60 and 67 for reference method and PT-μSPE, respectively). This is because the techniques and sample throughput as well as the sample volume are consistent for these methods. A higher score is obtained for the direct injection method (85.0). This difference is related to the elimination of the sample preparation step (point 5) and the resulting larger number of samples that can be prepared simultaneously (point 4), as well as the use of ethanol only, a very common commercially available solvent (point 7).

As already mentioned, GHG emission evaluation, AGREE, and BAGI provide information on selected aspects of a method, but they do not allow a global evaluation of the performance of the developed methods. Therefore, the RGB method was applied to the investigated methods [22]. First, the criteria for each attribute (red/green/blue), their weighting (w) in the overall evaluation and the reference values were selected. Since the aim of this study was to improve the greenness of the analysis, with accurate results and reliable analytical performance, a higher relative weight (*W* = 6) was assigned to the green and red sections, while a relative weight of 4 was assigned to the blue section. The validation parameters were used to evaluate the analytical performance (red attribute) of the four methods. In particular, the attributes considered were (i) relative errors, (ii) experimentally measured LOD and (iii) LOQ, (iv) linearity, (v) repeatability, and (vi) reproducibility. A relative weight of *w* = 1 was assigned for all criteria, except for relative error, which was assigned a weight of 5 as this parameter is a measure of the accuracy of the method. Regarding the greenness of the methods, the highest importance (*w* = 4) was given to the energy consumption for each analysis (kWh), taking into account all equipment used for the analysis (GC, MS, autosampler, PC, hydrogen generator). Another important point for GC analysis is the use of helium as carrier gas: the consumption of non-renewable gas or gas produced from non-renewable sources should be minimized or suppressed to reduce the environmental footprint of the analyses. The second criterion for the green parameter was therefore the amount of non-renewable materials, including carrier gas, used for 10 analyses (*w* = 2). The third criterion was the consumption of reagents (excluding carrier gas) used every 15 analyses or 5 samples (*w* = 3), which includes all materials used in sample preparation. The last “green” criterion involved the toxicity of the chemicals used (*w* = 1). A hazard rating was assigned to each reagent, taking into account both the number of hazards and their category according to the European Regulation (EC) No. 1272/2008 [46]. The overall hazard score is obtained by multiplying the number of hazards in category I by 1, those in category II by 0.8, those in category III by 0.6 and those in category IV by 0.4. A similar approach was proposed by Nowak and coworkers [47]. Finally, the practical and economic aspects of the methods were considered for the calculation of the blueness. Energy and gas consumption were considered for the cost of materials (weight of 3), as these are the items that change the most depending on the method used. It is important to note that the calculation on energy consumption was based on a time frame of 24 h. Nevertheless, the number of analyses was set at 15, as a reduction in analysis time should not be accompanied by an increase in the number of analyses. The time efficiency, i.e., the number of samples analyzed in 24 h, was used for productivity. The practical effectiveness of a method is another important aspect, particularly in the context of quality control. The blue attribute also takes into account the need for specialized or advanced instruments and the operator’s ability to manage the analytical system (requirements) and finally the maintenance of the instruments. Assigning a scale for scoring each criterion is another important step in creating the template. Novak et al. [23] suggest assigning the Low Acceptable Value (LAV), a value above which the results may be acceptable, with a score of 33.3 on a scale of 1–100, and the Low Satisfactory Value (LSV), a value above which the results may be considered satisfactory, with a score of 66.6. In addition to these two reference values, we have introduced a new value, the Highest Satisfactory Value (HSV) [48], which represents the optimal value to be achieved with a score of 100. In this work, the minimum tolerated values indicated in the official regulations for the validation of the analytical method [31] were chosen for the redness section as LAV, while the HSV was chosen considering the optimal results that can be obtained for each validation parameter. LSV was thereby considered as the intermediate value between LAV and HSV. For the greenness and blueness sections, the consumption associated with the GC analysis was calculated by setting the HSV to the lowest possible energy and gas carrier (hydrogen) consumption achieved when the device is simply switched to standby mode.

**Fig. 3:**
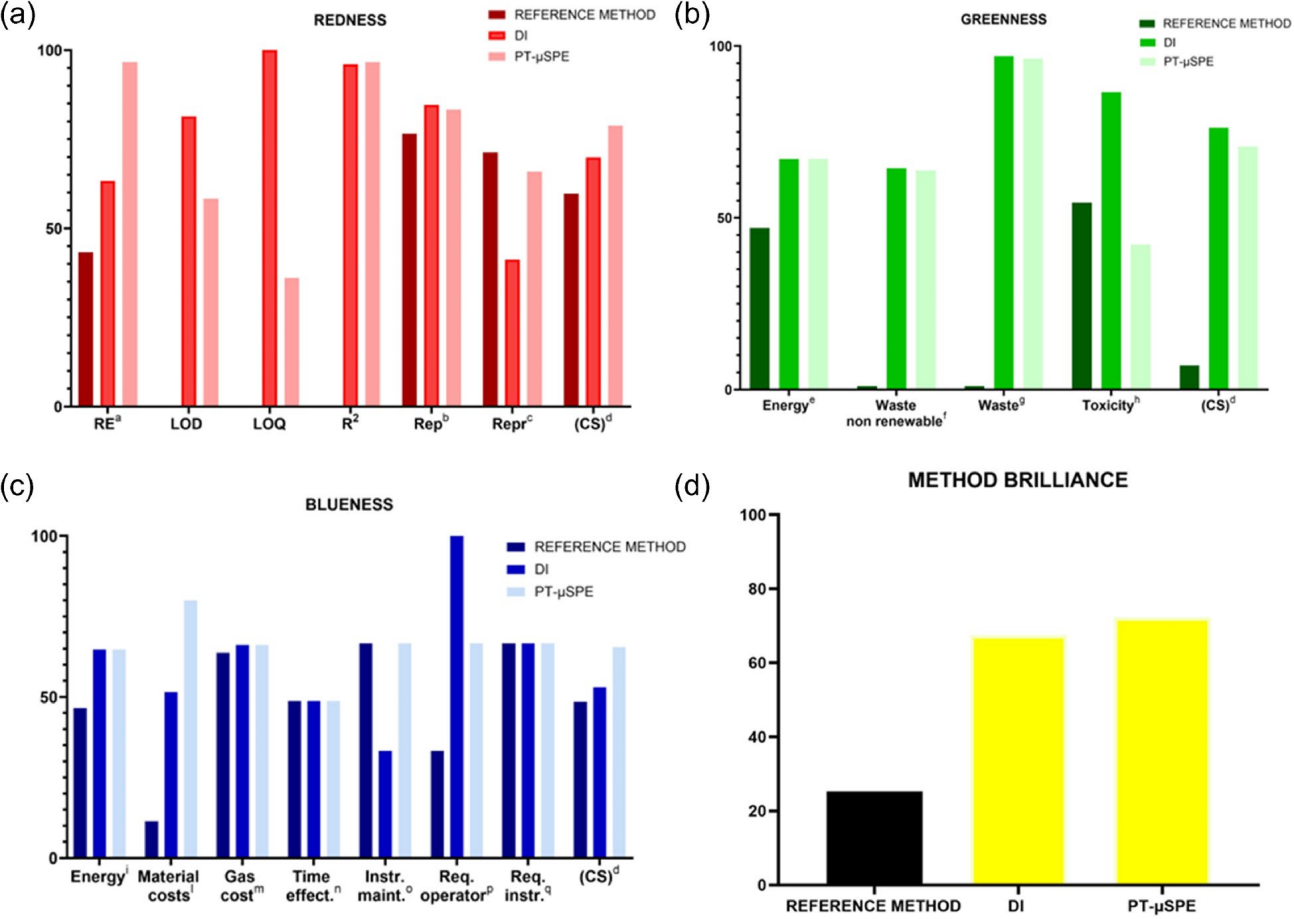
Comparison of RGB results for the reference SPE, direct injection, and optimized PT-µSPE methods for the redness (**a**), greenness (**b**), blueness (**c**), and method brilliance (**d**). The color of the bars in the method brilliance (panel (**d**)) reflect the final color of the method obtained with the RGB evaluation. Redness: ^a^relative errors, ^b^repeatability, ^c^reproducibility, ^d^color score; Greenness: ^e^energy consumption/analysis, ^f^amount of reagent, waste non-renewable/10 analysis; ^g^amount of reagent/waste (g/10 analyses), ^h^toxicity of reagents; Blueness: ^i^energy consumption, ^l^material costs, ^m^gas consumption cost, ^n^time effectiveness, ^o^instrumental maintenance, ^p^requirements operator, ^q^requirements instrument

The RGB results for the three methods investigated in this study are summarized in Fig. 3 and reported in detail in the supplementary materials in Figure S7. The overall evaluation of the reference method shows a method brilliance (MB) of 25.3%, because the three attributes are not optimal, especially the greenness. As previously highlighted, the low score in terms of greenness (7.0%) is mainly due to the high energy consumption resulting from the need for separate analyses to obtain quantitative and semi-quantitative results, as well as the use of a higher amount of toxic and/or not renewable materials and reagents. The blueness score was only 48.5%, again due to the high energy and material consumption. The reference method was not validated as it was not considered optimal from the beginning and therefore only repeatability, reproducibility, and relative errors were considered for the evaluation of the analytical performance. A result of 59.7% was achieved for the red section. The direct injection method does not require any sample preparation and only ethanol is used as diluent solvent. An improvement in energy and gas consumption and time efficiency was observed for the blue attribute, as well as for the greenness section due to the adoption of a single analysis with a simultaneous MS and FID detection. However, also in this case, the analytical performance of the methods is not satisfactory (color score 69.9%) because of the low scores obtained in terms of relative errors and reproducibility. Finally, the developed PT-µSPE method shows a more balanced performance in terms of the three attributes and the MB is 72.3%. Compared to the reference method, the analytical performance is improved, and the greenness attribute increased to 70.7%, not only because of the improvements related to the single analysis with the dual detection (with a passive splitter that does not require additional flow controllers), but also because a lower amount of materials is used, which also affects the cost of the analysis. The simplicity of the method is also underlined by the requirements for the operator, which can be considered moderate, as only good knowledge of micropipette handling is required. In general, the overall evaluation of the four methods shows that the PT-µSPE method provides the most accurate results with the least impact on the environment due to sample preparation and an acceptable productivity.

The sustainability of the proposed methods was then assessed using the NQS (Need, Quality, Sustainability) index created by Kiwfo et al. [26] in 2023. As mentioned in the “Introduction”, this tool evaluates the analytical method in terms of Need, i.e., the actual need for analytical methods, Quality, represented by the WAC, and Sustainability, i.e., compliance with the Sustainable Development Goal [28]. The results (reported in Figure S8) show a higher NQS index for the direct injection method (60), which is due to the absence of sample preparation and the use of non-toxic reagents leads to a need and quality score of 75. The PT-µSPE (Figure S8c) shows a slightly lower NQS index (58) and could therefore be used as an alternative, while the reference method (Figure S8a) shows a drastically lower final index of 23. All methods fail to a high degree in terms of sustainability. This is the parameter that deserves more attention in future work. As mentioned earlier, the quality of the methods is determined based on the whiteness parameter by using the White Analytical Chemistry (WAC) metric tool [24]. The results (illustrated in Figure S9a-c) show a similar trend compared to RGB, although the higher flexibility of the latter allows a more holistic view of the methods, highlighting the better characteristics in terms of accuracy of the results of the developed PT-µSPE method compared to the direct injection approach. On the opposite, this last is characterized by a better greenness value performance and appears as a better alternative when evaluated with the WAC and NQS metric tools.

## Conclusions

In this study, a pipette-tip micro-solid-phase extraction (PT-µSPE) GC–MS/FID method was successfully developed and validated for the analysis of water-based perfumes containing a considerable amount of water and cosmetic agents. The proposed method showed clear advantages over conventional methods in terms of environmental compatibility, efficiency, and overall performance.

Compared to reference extraction methods, PT-µSPE significantly reduces the amount of materials and solvents required, complies with the principles of Green Analytical Chemistry (GAC) and minimizes the environmental impact. In addition, the simultaneous FID and MS detection used for semi-quantitative and quantitative purposes, respectively, enables a drastic reduction in energy and gas consumption, in addition to saving time. The method also demonstrated robust analytical performance, providing accurate quantitative and semi-quantitative data with high repeatability, reproducibility, and minimal matrix effects. Furthermore, the method was successfully applied to commercial samples, confirming its suitability for routine quality control in the fragrance industry.

The PT-µSPE GC–MS/FID method was evaluated for its greenness and overall performance using various metric tools, including RGB, WAC, and NQS. Although these approaches only allow a semi-quantitative comparison between different methods, the assessments confirmed that the PT-µSPE method offers a balanced performance in terms of environmental, practical and analytical criteria, making it a superior alternative for routine analysis of complex perfumes. Future improvements should focus on further increasing the sustainability of the method by exploring less toxic and more renewable materials, also with a view to better aligning with the Sustainable Development Goals, and to quantitatively and objectively estimate the impact on the environment (for instance in terms of global warming) through appropriate approaches such as life cycle assessment (LCA).

## Supplementary Information

Below is the link to the electronic supplementary material.

Supplementary file1 (DOCX 724 KB)

## Data Availability

The datasets generated during the current study are available from the corresponding author on reasonable request.
