# Supplementary material for: Development, validation, and assessment of a reliable and white method for the analysis of water-based perfumes by pipette-tip micro-solid-phase extraction combined with dual detection gas chromatography
Source: Anal Bioanal Chem. 2025 May 7;417(16):3579–96. doi: 10.1007/s00216-025-05889-x (PMC12206168; doi:10.1007/s00216-025-05889-x)
Supplement: Supplementary file 1 — Supplementary file1 (DOCX 724 KB) [file 216_2025_5889_MOESM1_ESM.docx]

**Supplementary Information**

**DEVELOPMENT, VALIDATION AND ASSESSMENT OF A RELIABLE AND WHITE METHOD FOR THE ANALYSIS OF WATER-BASED PERFUMES BY PIPETTE-TIP MICRO-SOLID-PHASE EXTRACTION COMBINED WITH DUAL DETECTION GAS CHROMATOGRAPHY**

*Gaia Bechis^1^, Giulia Quaranta^1^, Carlo Bicchi^1^, Arianna Marengo^1^, Barbara Sgorbini^1^, Patrizia Rubiolo^1^,*

*Cecilia Cagliero^1^*

*^1^Department of Drug Science and Technology, University of Turin, 10125 Turin, Italy*

Table S1…………………………………………………………………………….Page S2

Table S2…………………………………………………………………………….Page S3

Table S3…………………………………………………………………………….Page S4

Table S4…………………………………………………………………………….Page S5

Table S5…………………………………………………………………………….Page S6

Table S6…………………………………………………………………………….Page S7

Figure S1……………………………………………………………………………Page S8

Figure S2……………………………………………………………………………Page S9

Figure S3……………………………………………………………………………Page S10

Figure S4……………………………………………………………………………Page S11

Figure S5……………………………………………………………………………Page S12

Figure S6……………………………………………………………………………Page S13

Figure S7……………………………………………………………………………Page S14

Figure S8……………………………………………………………………………Page S15

Figure S9……………………………………………………………………………Page S16

***Address for correspondance**

Cecilia Cagliero

Department of Drug Science and Technology, University of Turin, Via Pietro Giuria 9, Torino, I-10125

Tel.: +39 011 6707133, e-mail address: [cecilia.cagliero@unito.it](mailto:cecilia.cagliero@unito.it), ORCID: 0000-0003-3512-6124

**Table S1.** SIM acquisition details for each compound. Suspected allergens are reported in bold. Legend. *I*^T^: linear retention index, *present in Perfumes with fragrance concentrate A and/or B. Reference for literature *I*^T^s: https://webbook.nist.gov/chemistry/

| **Window** | **Time (min)** | **INCI name** | **Common name** | **Supplier** | **CAS number** | ***I^T^* literature** | ***I^T^* experimental** | **Quantified ion** | **Qualifier ion 1** | **Qualifier ion 2** | **Fragrance concentrate*** |
| --- | --- | --- | --- | --- | --- | --- | --- | --- | --- | --- | --- |
| **SIM1** | 2.70-11.0 | **Benzaldehyde** | **Benzaldehyde** | Merck | 100-52-7 | 971 | 971 | 77 | 105 | 106 | A |
| **SIM2** | 11.0-16.0 | **Limonene** | **Limonene** | Merck | 138-86-3 | 1029 | 1034 | 68 | 93 | 67 | B |
| **SIM3** | 16.0-21.0 | Ethyl linalool 1 | Ethyl linalool 1 | Givaudan | 10339-55-6 | 1183 | 1184 | 55 | 71 | 93 | B |
|  |  | Ethyl linalool 2 | Ethyl linalool 2 | Givaudan |  | 1196 | 1197 |  |  |  | B |
|  |  | 1,4-dibromo benzene | 1,4-dibromo benzene | Merck | 106-37-6 | 1193 | 1208 | 236 | 234 | 238 | Internal standard |
|  |  | **Linalyl acetate** | **Linalyl acetate** | Merck | 115-95-7 | 1257 | 1251 | 93 | 121 | 80 | B |
| **SIM4** | 21.0-28.0 | ***trans-β-*caryophyllene** | ***trans*-β-caryophyllene** | Merck | 87-44-5 | 1423 | 1431 | 93 | 91 | 133 | B |
|  |  | **Coumarin** | **Coumarin** | Merck | 91-64-5 | 1456 | 1457 | 118 | 146 | 90 | A |
| **SIM5** | 28.0-36.5 | Methylenedioxyphenyl methylpropanal | Helional | Merck | 1205-17-0 | 1579 | 1580 | 135 | 77 | 192 | B |
|  |  | **Tetramethyl acetyloctahydronaphthalenes 1** | **Iso e super 1** | Merck | 54464-57-2 | 1671 | 1673 | 191 | 121 | 109 | A/B |
|  |  | **Tetramethyl acetyloctahydronaphthalenes 2** | **Iso e super 2** | Merck |  | 1677 | 1678 |  |  |  | A/B |
|  |  | **Tetramethyl acetyloctahydronaphthalenes 3** | **Iso e super 3** | Merck |  | 1680 | 1681 |  |  |  | A/B |
| **SIM 6** | 36.5-37.7 | **Benzyl benzoate** | **Benzyl benzoate** | Merck | 120-51-4 | 1775 | 1786 | 105 | 91 | 212 | A/B |
|  |  | Ambroxide | Ambroxide | Symrise | 6790-58-5 | 1785 | 1786 | 221 | 137 | 97 | A/B |
| **SIM7** | 37.7-40.5 | Oxacyclohexadecenone, (12E) 1 | Habanolide 1 | Firmenich | 111879-80-2 | 1834 | 1835 | 68 | 81 | 95 | A |
|  |  | Oxacyclohexadecenone, (12E) 2 | Habanolide 2 | Firmenich |  | 1839 | 1840 |  |  |  | A |
|  |  | Oxacyclohexadecenone, (12E) 3 | Habanolide 3 | Firmenich |  | 1846 | 1848 |  |  |  | A |
|  |  | 1,3,4,6,7,8-Hexahydro-4,6,6,7,8,8-hexamethylcyclopenta-γ-2-benzopyran | Galaxolide | Merck | 1222-05-5 | 1847 | 1854 | 243 | 258 | 213 | B |
| **SIM8** | 40.5-50.62 | Oxacycloheptadec-10-en-2-one | Ambrettolide | Merck | 28645-51-4 | 1925 | 1945 | 67 | 82 | 96 | A |
|  |  | 4,4'-dibromobphenyl | 4,4'-dibromobphenyl | Merck | 92-86-4 | 2033 | 2035 | 312 | 310 | 152 | Internal standard |

**Table S2.** Ingredients of the commercial Perfumes analysed (excluding allergens, antioxidants, dyes). Cosmetic agents are reported in bold. *Fragrance concentrate: perfumes characterized by the same fragrance raw materials.

| **Samples** | **Composition** | **CAS cosmetic agent** | **Log P cosmetic agents** |
| --- | --- | --- | --- |
| Perfume 1 | Alcohol, Water, ***Glycerin***, Fragrance concentrate | 56-81-5 | -1.8 |
| Perfume 3 |  |  |  |
| Perfume 5 |  |  |  |
| Perfume 7 |  |  |  |
| Perfume 2 | Alcohol, Water, Fragrance concentrate, ***PEG-40 hydrogenated castor oil*** | 61788-85-0 | -- |
| Perfume 4 |  |  |  |
| Perfume 6 |  |  |  |
| Perfume 8 |  |  |  |

**Table S3**. Extraction conditions tested

| **Method** | **Amount of**  **sample** | **Type of**  **sorbent** | **Amount of sorbent** | **Type of**  **extraction solvent** | **Amount of**  **extraction solvent** |
| --- | --- | --- | --- | --- | --- |
| REFERENCE | 4.5 g | EXtrelut^®^ NT | 2.5 g | Cyclohexane | 12.5 g |
| TEST 1 | 2.5 g | EXtrelut^®^ NT | 2.5 g | Cyclohexane | 7.0 g |
| TEST 2 | 1.0 g | EXtrelut^®^ NT | 1.0 g | Cyclohexane | 2.5 g |
| TEST 3 | 1.0 g | Celite 545^®^ | 1.0 g | Cyclohexane | 2.5 g |
| TEST 4 | 100 mg | Celite 545^®^ | 100 mg | Cyclohexane | 500 mg |
| TEST 5 | 100 mg | Celite 545^®^ | 100 mg | Heptane | 500 mg |
| TEST 6 | 100 mg | Celite 545^®^ | 100 mg | Heptane/Ethyl acetate (50:50) | 500 mg |
| TEST 7 | 100 mg | Celite 545^®^ | 100 mg | Heptane/Acetonitrile (50:50) | 500 mg |
| TEST 8 | 100 mg | Celite 545^®^ | 100 mg | Heptane/Ethyl acetate (70:30) | 500 mg |
| TEST 9 | 100 mg | Celite 545^®^ | 100 mg | Heptane/Acetonitrile (70:30) | 500 mg |
| PT-µSPE | 10 mg | Celite 545^®^ | 10 mg | Heptane/Ethyl acetate (70:30) | 100 mg |

**Table S4.** Analytical figure of merits of the PT-µSPE and direct injection GC-MS/FID methods for the investigated analytes.

| **Compounds** | **Linearity (R^2^)**  **Range 1-50 mg kg^-1^** | **Linearity (R^2^) Range 100-500 mg kg^-1^** | **LOD**  **(mg kg^-1^)** | **LOQ**  **(mg kg^-1^)** |
| --- | --- | --- | --- | --- |
| **Direct injection** | | | | |
| Limonene | 0.995 | 0.995 | <0.01 | <0.01 |
| Ethyl linalool 1 | 0.990 | 0.997 | 0.20 | 1.95 |
| Ethyl linalool 2 | 0.994 | 0.997 | 0.06 | 0.61 |
| Iso e super 1 | 0.996 | 0.996 | 0.08 | 0.10 |
| Iso e super 2 | 0.994 | 0.996 | 0.03 | 0.08 |
| Iso e super 3 | 0.993 | 0.997 | 0.03 | 0.14 |
| Ambroxide | 0.996 | 0.997 | 0.01 | 0.10 |
| Benzyl benzoate | 0.995 | 0.992 | 0.01 | 0.03 |
| Galaxolide | 0.995 | 0.991 | 0.02 | 0.10 |
| Linalyl acetate | 0.998 | 0.981 | 0.01 | 0.03 |
| *trans*-β***-***caryophyllene | 0.998 | 0.991 | 0.10 | 0.20 |
| Helional | 0.994 | 0.991 | 0.04 | 0.50 |
| Benzaldehyde | 0.993 | 0.996 | 0.10 | 0.50 |
| Habanolide 1 | 0.991 | 0.990 | 0.04 | 0.05 |
| Habanolide 2 | 0.988 | 0.988 | 0.05 | 0.29 |
| Habanolide 3 | 0.988 | 0.988 | 0.06 | 0.14 |
| Ambrettolide | 0.992 | 0.993 | 0.10 | 0.20 |
| Coumarin | 0.995 | 0.995 | 0.04 | 0.10 |
| **PT-µSPE** | | | | |
| Limonene | 0.994 | 0.997 | <0.01 | 0.07 |
| Ethyl linalool 1 | 0.988 | 0.999 | 0.20 | 2.63 |
| Ethyl linalool 2 | 0.995 | 0.999 | 0.30 | 3.05 |
| Iso e super 1 | 0.994 | 0.992 | 0.02 | 0.38 |
| Iso e super 2 | 0.996 | 0.998 | 0.07 | 0.33 |
| Iso e super 3 | 0.996 | 0.993 | 0.01 | 0.08 |
| Ambroxide | 0.991 | 0.993 | 0.20 | 2.46 |
| Galaxolide | 0.992 | 0.991 | 0.10 | 0.50 |
| Linalyl acetate | 0.998 | 1.000 | 0.10 | 0.50 |
| *trans*-β***-***caryophyllene | 0.997 | 0.998 | 0.50 | 1.00 |
| Helional | 0.997 | 0.999 | 0.30 | 0.50 |
| Benzyl benzoate | 0.997 | 0.997 | 0.10 | 0.50 |
| Benzaldehyde | 0.988 | 0.998 | 0.50 | 3.30 |
| Habanolide 1 | 0.995 | 0.993 | 0.29 | 1.50 |
| Habanolide 2 | 0.999 | 0.993 | 0.20 | 2.93 |
| Habanolide 3 | 0.995 | 0.993 | 0.20 | 2.85 |
| Ambrettolide | 0.997 | 0.990 | 0.50 | 2.00 |
| Coumarin | 0.998 | 0.997 | 0.50 | 2.05 |

**Table S5.** Relative percentage composition of target compounds in the investigated Perfumes after PT-µSPE (n=9). Relative errors, compared to the direct injection, are reported in brackets.

| **Compounds** | **Relative composition (%) ± standard deviation (*relative errors*)** | | | |
| --- | --- | --- | --- | --- |
|  | **PT-µSPE Perfume 1** | **PT-µSPE Perfume 2** | **PT-µSPE Perfume 3** | **PT-µSPE Perfume 4** |
| Benzaldehyde | 1.0±0.03 (*0.01*) | 1.3±0.03 (-*0.34*) | 0.9±0.05 (*0.07*) | 0.8±0.06 (*0.04*) |
| Coumarin | 5.7±0.00 (*0.01*) | 6.2±0.05 (-*0.09*) | 6.3±0.01 (-*0.11*) | 6.2±0.02 (0.04) |
| Iso e super 1 | 11.3±0.00 (-*0.06*) | 12.1±0.01 (-*0.13*) | 10.2±0.01 (*0.04*) | 10.3±0.01 (-*0.01*) |
| Iso e super 2 | 1.2±0.02 (-*0.13*) | 1.6±0.02 (-*0.46*) | 1.3±0.02 (-*0.23*) | 1.3±0.02 (-*0.03*) |
| Iso e super 3 | 2.5±0.02 (-*0.20*) | 2.7±0.01 (-*0.37*) | 2.3±0.02 (-*0.17*) | 2.3±0.01 (-*0.06*) |
| Ambroxide | 1.6±0.01 (-*0.18*) | 1.9±0.00 (-*0.44*) | 1.7±0.01 (-*0.29*) | 1.7±0.03 (-*0.04*) |
| Benzyl benzoate |  |  |  |  |
| Habanolide 1 | 5.2±0.01 (-*0.12*) | 5.4±0.01 (-*0.17)* | 5.3±0.01 (-*0.15*) | 5.2±0.01 (-*0.08*) |
| Habanolide 2 | 3.6±0.01 (-*0.19*) | 3.9±0.01 (-*0.28*) | 3.7±0.02 (-*0.20*) | 3.6±0.01 (-*0.09*) |
| Habanolide 3 | 3.5±0.01 (-*0.19*) | 3.7±0.02 (-*0.26*) | 3.6±0.01 (-*0.22*) | 3.5±0.02 (-*0.09*) |
| Ambrettolide | 1.4±0.01 (-*0.12*) | 1.5±0.04 (-*0.24*) | 1.6±0.04 (-*0.33*) | 1.6±0.02 (-*0.12*) |
|  | **PT-µSPE Perfume 5** | **PT-µSPE Perfume 6** | **PT-µSPE Perfume 7** | **PT-µSPE Perfume 8** |
| Limonene | 6.6±0.01 (-*0.02*) | 5.1±0.11 (*0.21*) | 3.0±0.06 (*0.54*) | 4.5±0.06 (*0.30*) |
| Ethyl linalool 1 | 1.7±0.02 (-*0.15*) | 1.6±0.03 (-*0.10*) | 1.3±0.05 (*0.12*) | 1.3±0.03 (*0.13*) |
| Ethyl linalool 2 | 2.7±0.02 (-*0.18*) | 2.6±0.02 (-*0.12*) | 2.0±0.05 (*0.13*) | 2.0±0.04 (*0.13*) |
| Iso e super 1 | 21.1±0.00 (*0.04*) | 22.5±0.00 (-*0.02*) | 22.8±0.00 (-*0.03*) | 22.9±0.01 (-*0.04*) |
| Iso e super 2 | 2.6±0.02 (-*0.07*) | 2.8±0.01 (-*0.18*) | 2.7±0.01 (-*0.11*) | 2.6±0.01 (-*0.08*) |
| Iso e super 3 | 4.6±0.01 (-*0.06*) | 4.8±0.00 (-*0.11*) | 4.8±0.01 (-*0.11*) | 4.7±0.01 (-*0.08*) |
| Ambroxide | 2.1±0.01 (-*0.07*) | 2.2±0.03 (-*0.10*) | 2.3±0.01 (-*0.19*) | 2.2±0.02 (-*0.13*) |
| Benzyl benzoate |  |  |  |  |
| Galaxolide | 10.1±0.01 (*0.04*) | 10.7±0.01 (-*0.01*) | 12.1±0.02 (-*0.14*) | 11.8±0.01 (-*0.12*) |
| Linalyl acetate | 3.9±0.04 (-*0.06*) | 3.6±0.00 (*0.02*) | 3.0±0.06 (*0.18*) | 3.1±0.03 (*0.16*) |
| *trans-β-*caryophyllene | 2.1±0.01 (-*0.05*) | 1.8±0.00 (*0.08*) | 1.6±0.04 (*0.18*) | 1.6±0.03 (*0.21*) |
| Helional | 0.9±0.03 (-*0.09*) | 1.0±0.09 (-*0.22*) | 1.0±0.04 (-*0.27*) | 1.0±0.02 (-*0.19*) |

**Table S6.** Uncertainty of the results calculated according to EN ISO/IEC 17025:2005 relative to the target compounds in the perfumes obtained by PT-µSPE and directly injected. Legend: u_r_ - Standard Uncertainty due to Repeatability, u_c_ - Combined Standard Uncertainty, U - Expanded Uncertainty.

| **Compounds** | **PT-µSPE Perfume 1** | | | **PT-µSPE Perfume 2** | | | **PT-µSPE Perfume 3** | | | **PT-µSPE Perfume 4** | | | **DI Perfume 1** | | | **DI Perfume 2** | | | **DI Perfume 3** | | | **DI Perfume 4** | | |
| --- | --- | --- | --- | --- | --- | --- | --- | --- | --- | --- | --- | --- | --- | --- | --- | --- | --- | --- | --- | --- | --- | --- | --- | --- |
|  | **u_r_** | **u_c_** | **U** | **u_r_** | **u_c_** | **U** | **u_r_** | **u_c_** | **U** | **u_r_** | **u_c_** | **Uz** | **u_r_** | **u_c_** | **U** | **u_r_** | **u_c_** | **U** | **u_r_** | **u_c_** | **U** | **u_r_** | **u_c_** | **U** |
| **Benzaldehyde** | 0.00912 | 0.159 | 0.32 | 0.01028 | 0.183 | 0.37 | 0.00007 | 0.006 | 0.012 | 0.00006 | 0.009 | 0.017 | 0.00035 | 1.569 | 3.1 | 0.00041 | 1.875 | 3.8 | 0.00369 | 0.040 | 0.079 | 0.00000 | 0.080 | 0.16 |
| **Coumarin** | 0.00357 | 2.394 | 4.8 | 0.01795 | 3.195 | 6.4 | 0.00111 | 0.120 | 0.24 | 0.00124 | 0.133 | 0.27 | 0.02060 | 4.408 | 8.8 | 0.02504 | 5.015 | 10 | 0.00018 | 0.123 | 0.25 | 0.00090 | 0.222 | 0.44 |
| **Iso e super 1** | 0.00701 | 3.458 | 6.9 | 0.02133 | 4.142 | 8.3 | 0.00083 | 0.140 | 0.28 | 0.00219 | 0.175 | 0.35 | 0.01076 | 5.360 | 10 | 0.00355 | 5.629 | 11 | 0.00042 | 0.174 | 0.35 | 0.00059 | 0.325 | 0.65 |
| **Iso e super 2** | 0.00133 | 0.379 | 0.76 | 0.00510 | 0.450 | 0.90 | 0.00011 | 0.020 | 0.040 | 0.00025 | 0.020 | 0.040 | 0.00032 | 0.621 | 1.2 | 0.00069 | 0.805 | 1.6 | 0.00011 | 0.022 | 0.045 | 0.00013 | 0.044 | 0.088 |
| **Iso e super 3** | 0.00108 | 0.656 | 1.3 | 0.00158 | 0.754 | 1.5 | 0.00024 | 0.031 | 0.062 | 0.00049 | 0.034 | 0.069 | 0.00244 | 1.011 | 2.0 | 0.00077 | 1.093 | 2.2 | 0.00004 | 0.038 | 0.076 | 0.00030 | 0.075 | 0.15 |
| **Ambroxide** | 0.00091 | 0.377 | 0.75 | 0.00588 | 0.394 | 0.79 | 0.00026 | 0.035 | 0.070 | 0.00042 | 0.019 | 0.039 | 0.00471 | 1.035 | 2.1 | 0.00267 | 1.103 | 2.2 | 0.00013 | 0.045 | 0.090 | 0.00071 | 0.093 | 0.19 |
| **Benzyl benzoate** | 0.00015 | 0.159 | 0.32 | 0.00020 | 0.183 | 0.37 | 0.00006 | 0.006 | 0.012 | 0.00004 | 0.009 | 0.017 | 0.00014 | 0.292 | 0.58 | 0.00024 | 0.311 | 0.62 | 0.00001 | 0.006 | 0.012 | 0.00005 | 0.012 | 0.024 |
| **Habanolide 1** | 0.00393 | 2.020 | 4.0 | 0.00460 | 2.354 | 4.7 | 0.00037 | 0.050 | 0.10 | 0.00201 | 0.106 | 0.21 | 0.03761 | 4.542 | 9.1 | 0.00047 | 4.142 | 8.3 | 0.00042 | 0.095 | 0.19 | 0.00133 | 0.175 | 0.35 |
| **Habanolide 2** | 0.00277 | 1.345 | 2.7 | 0.00453 | 1.601 | 3.2 | 0.00034 | 0.035 | 0.070 | 0.00145 | 0.072 | 0.14 | 0.02510 | 2.786 | 5.2 | 0.00011 | 2.775 | 5.5 | 0.00000 | 0.067 | 0.13 | 0.00014 | 0.123 | 0.25 |
| **Habanolide 3** | 0.00252 | 1.271 | 2.5 | 0.00569 | 1.529 | 3.1 | 0.00039 | 0.034 | 0.068 | 0.00121 | 0.068 | 0.14 | 0.05678 | 3.467 | 6.9 | 0.00020 | 2.698 | 5.4 | 0.00018 | 0.064 | 0.13 | 0.00002 | 0.117 | 0.23 |
| **Ambrettolide** | 0.00150 | 0.517 | 1.0 | 0.00173 | 0.609 | 1.2 | 0.00010 | 0.020 | 0.040 | 0.00035 | 0.028 | 0.057 | 0.00049 | 1.041 | 2.1 | 0.00063 | 1.111 | 2.2 | 0.00008 | 0.026 | 0.053 | 0.00015 | 0.049 | 0.099 |
|  | **PT-µSPE Perfume 5** | | | **PT-µSPE Perfume 6** | | | **PT-µSPE Perfume 7** | | | **PT-µSPE Perfume 8** | | | **DI Perfume 5** | | | **DI Perfume 6** | | | **DI Perfume 7** | | | **DI Perfume 8** | | |
|  | **u_r_** | **u_c_** | **U** | **u_r_** | **u_c_** | **U** | **u_r_** | **u_c_** | **U** | **u_r_** | **u_c_** | **U** | **u_r_** | **u_c_** | **U** | **u_r_** | **u_c_** | **U** | **u_r_** | **u_c_** | **U** | **u_r_** | **u_c_** | **U** |
| **Limonene** | 0.21728 | 4.485 | 9.0 | 0.03202 | 3.701 | 7.4 | 0.00013 | 0.048 | 0.095 | 0.00052 | 0.048 | 0.095 | 0.00406 | 2.088 | 4.2 | 0.01324 | 2.706 | 5.4 | 0.00007 | 0.060 | 0.12 | 0.00000 | 0.145 | 0.29 |
| **Ethyl linalool 1** | 0.00641 | 0.906 | 1.8 | 0.00085 | 0.867 | 1.7 | 0.00001 | 0.025 | 0.051 | 0.00033 | 0.025 | 0.051 | 0.00122 | 0.924 | 1.8 | 0.00514 | 0.977 | 2.0 | 0.00002 | 0.035 | 0.069 | 0.00009 | 0.055 | 0.11 |
| **Ethyl linalool 2** | 0.00498 | 1.313 | 2.6 | 0.00166 | 1.320 | 2.6 | 0.00007 | 0.038 | 0.077 | 0.00057 | 0.038 | 0.077 | 0.00047 | 1.374 | 2.7 | 0.00557 | 1.494 | 3.0 | 0.00022 | 0.056 | 0.11 | 0.00008 | 0.087 | 0.17 |
| **Iso e super 1** | 0.13200 | 6.279 | 12 | 0.15060 | 8.333 | 16 | 0.00594 | 0.338 | 0.68 | 0.00424 | 0.496 | 0.99 | 0.03940 | 8.511 | 17 | 0.01116 | 10.662 | 21 | 0.00006 | 0.449 | 0.90 | 0.00047 | 0.351 | 0.70 |
| **Iso e super 2** | 0.00278 | 1.083 | 2.2 | 0.02298 | 1.265 | 2.5 | 0.00079 | 0.050 | 0.10 | 0.00042 | 0.076 | 0.15 | 0.01307 | 1.309 | 2.6 | 0.00022 | 1.300 | 2.6 | 0.00016 | 0.062 | 0.12 | 0.00001 | 0.099 | 0.20 |
| **Iso e super 3** | 0.02118 | 2.022 | 4.0 | 0.01637 | 1.972 | 3.9 | 0.00157 | 0.080 | 0.16 | 0.00183 | 0.122 | 0.24 | 0.01802 | 1.867 | 3.7 | 0.00434 | 2.157 | 4.3 | 0.00005 | 0.110 | 0.22 | 0.00016 | 0.166 | 0.33 |
| **Ambroxide** | 0.00492 | 0.622 | 1.2 | 0.00542 | 0.713 | 1.4 | 0.00071 | 0.040 | 0.080 | 0.00126 | 0.060 | 0.12 | 0.00693 | 0.964 | 1.9 | 0.00376 | 1.073 | 2.1 | 0.00016 | 0.057 | 0.11 | 0.00030 | 0.095 | 0.19 |
| **Benzyl benzoate** | 0.00113 | 0.401 | 0.80 | 0.01355 | 0.448 | 0.90 | 0.00019 | 0.015 | 0.029 | 0.00018 | 0.020 | 0.040 | 0.00632 | 0.687 | 1.4 | 0.00372 | 0.734 | 1.5 | 0.00003 | 0.021 | 0.042 | 0.00009 | 0.033 | 0.066 |
| **Galaxolide** | 0.08427 | 4.579 | 9.1 | 0.12392 | 6.158 | 12 | 0.00722 | 0.269 | 0.54 | 0.00473 | 0.399 | 0.80 | 0.02608 | 6.312 | 12 | 0.00553 | 7.499 | 15 | 0.00000 | 0.364 | 0.73 | 0.00270 | 0.546 | 1.1 |
| **Linalyl acetate** | 0.00152 | 1.573 | 3.1 | 0.23106 | 1.719 | 3.4 | 0.00002 | 0.031 | 0.061 | 0.00006 | 0.047 | 0.095 | 0.00061 | 1.563 | 3.1 | 0.00504 | 1.755 | 3.5 | 0.00002 | 0.050 | 0.10 | 0.00011 | 0.078 | 0.15 |
| **trans-β-caryophyllene** | 0.00061 | 0.931 | 1.9 | 0.13652 | 2.763 | 5.5 | 0.00000 | 0.020 | 0.041 | 0.00001 | 0.031 | 0.063 | 0.00030 | 1.015 | 2.0 | 0.00030 | 1.032 | 2.1 | 0.00005 | 0.032 | 0.063 | 0.00001 | 0.050 | 0.10 |
| **Helional** | 0.00085 | 0.486 | 0.97 | 0.02309 | 0.582 | 1.2 | 0.00015 | 0.017 | 0.034 | 0.00019 | 0.023 | 0.045 | 0.00819 | 0.963 | 1.9 | 0.00854 | 0.950 | 1.9 | 0.00000 | 0.027 | 0.053 | 0.00001 | 0.042 | 0.083 |


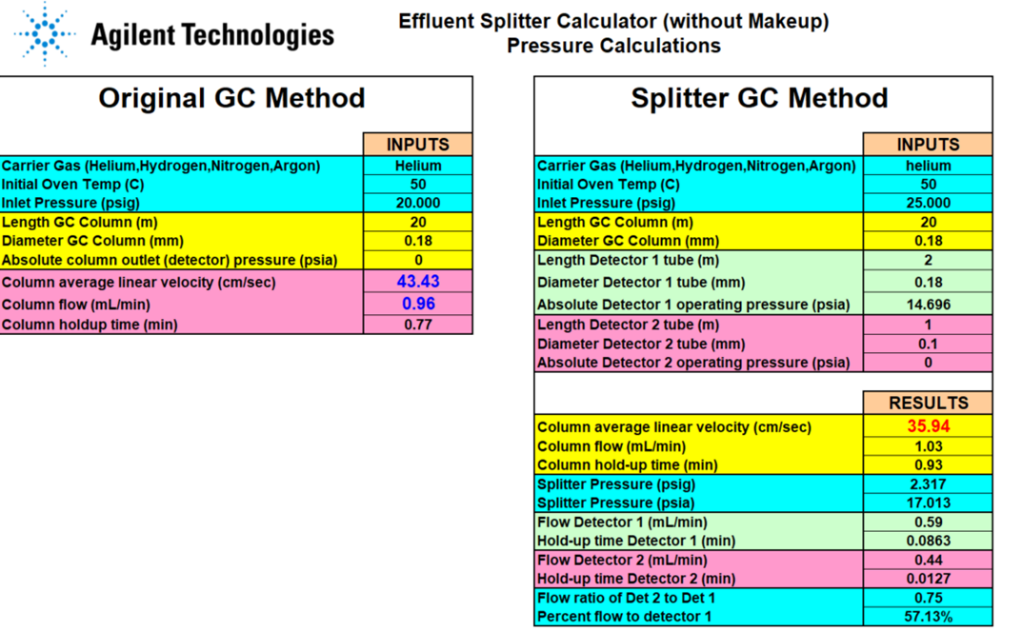


(b)

(a)


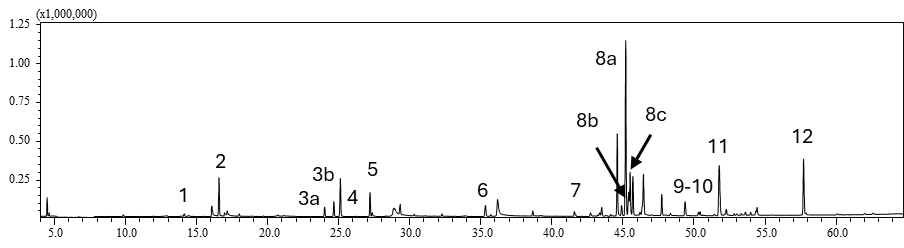


(c)


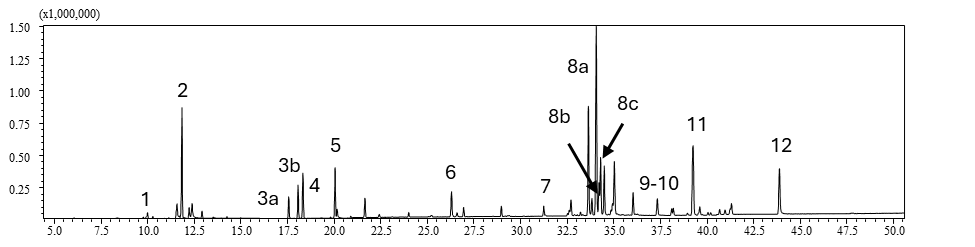


(d)


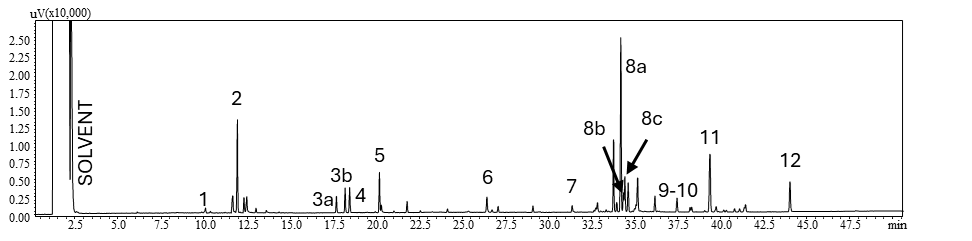


**Figure S1**. (a) Configuration and analytical conditions of the original GC-MS method and optimized GC-MS/FID method as calculated by the Effluent Splitter Pressure Calculator (Agilent) used to calculate the optimal capillary geometry, flow rate and pressure balance between the two detectors (MS and FID); (b) GC-MS profile of Perfume 5 obtained with single detection and (c) MS and (d) FID profiles obtained with the optimized GC-MS/FID approach. Analysis condition for (b): oven temperature from 50 °C to 280 °C at 3 °C min^-1^; pressure 54.2 kPa, flow 1.0 mL min^-1^; split ratio 25:1. Legend: 1. β-pinene, 2. limonene, 3a. ethyl linalool 1, 3b. ethyl linalool 2, 4. 1,4-dibromobenzene, 5. linalyl acetate, 6. ***trans-β-***caryophyllene, 7. helional, 8a. iso e super 1, 8b, iso e super 2, 8c, iso e super 3, 9. benzyl benzoate, 10. ambroxide, 11. galaxolide, 12. 4,4’-dibromobiphenyl.


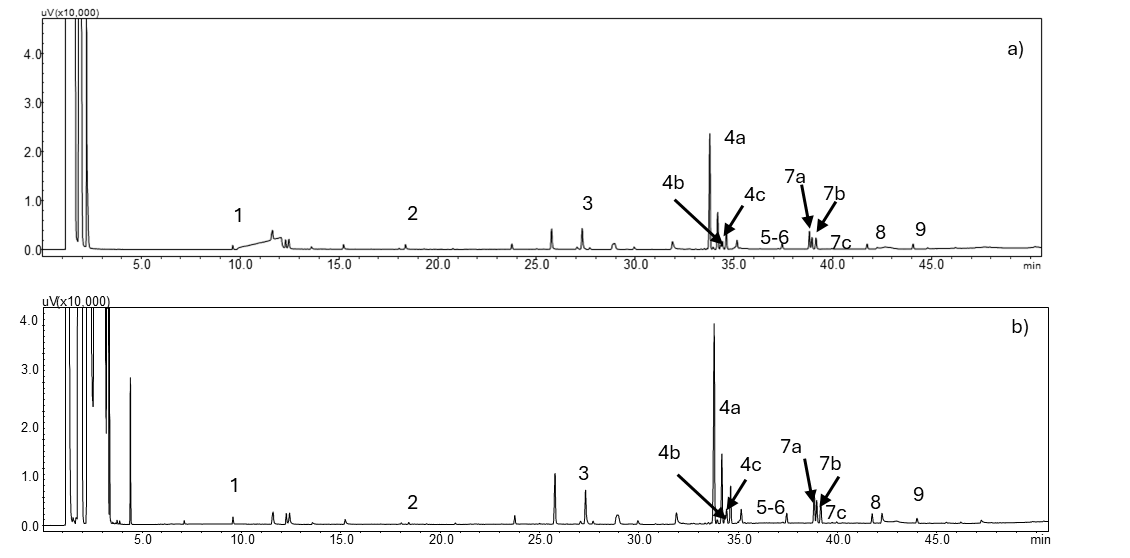


**Figure S2** Chromatographic profile of the Perfume 1 directly injected (a) after extraction with PT-µSPE (b). 1. benzaldehyde, 2. 1,4-dibromobenzene, 3. coumarin, 4a. iso e super 1, 4b. iso e super 2, 4c. iso e super 3, 5. benzyl benzoate, 6. amboxide, 7a. habanolide 1, 7b. habanolide 2, 7c. habanolide 3, 8. ambrettolide, 9. 4,4’-dibromobiphenyl. The red circle indicates the zone of elution of glycerin.

**
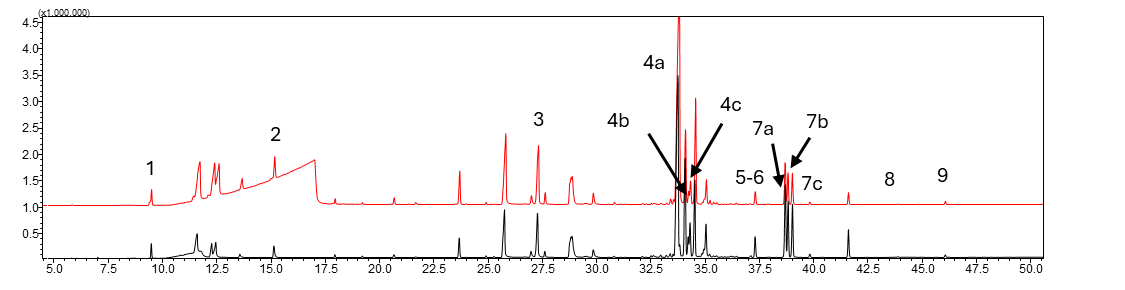
**

**Figure S3.** Chromatographic profile comparison of Perfume 5 after extraction with heptane/ethyl acetate (black) and heptane/acetonitrile (red). 1. benzaldehyde, 2. 1,4-dibromobenzene, 3. coumarin, 4a. iso e super 1, 4b. iso e super 2, 4c. iso e super 3, 5. benzyl benzoate, 6. amboxide, 7a. habanolide 1, 7b. habanolide 2, 7c. habanolide 3, 8. ambrettolide, 9. 4,4’-dibromobiphenyl. The red circle indicates the zone of elution of glycerin.

**
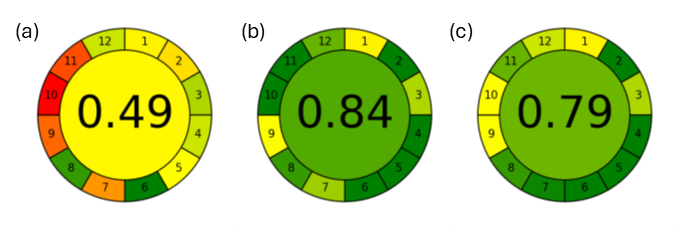
**

**Figure S4.** Comparison of AGREE results for the a) reference, b) direct injection and c) optimized PT-µSPE methods.

**a)**

**b)**

**Figure S5.** Comparison of a) greenhouse gas (GHG) emissions results for the reference, direct injection and optimized PT-µSPE methods expressed in terms of kgCO₂eq. b) GHG emission parameters considered. These results are based on data from ecoinvent 3.11 and characterized using the ReCiPe 2016 Midpoint (H) method.

**
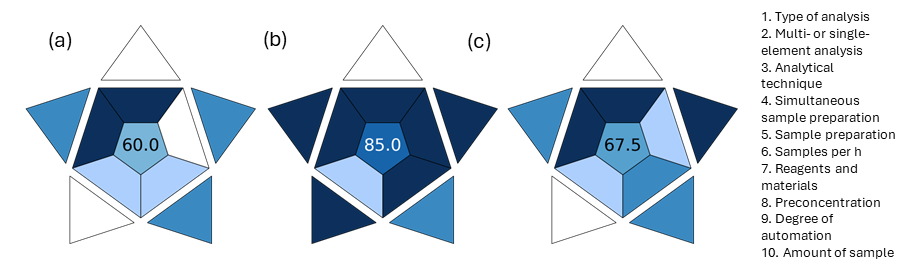
**

**Figure S6.** Comparison of Blue Applicability Grade Index (BAGI) results for the a) reference, b) direct injection and c) optimized PT-µSPE methods.

**a)**

**b)**

**c)**

**Figure S7.** Detailed results of Red-Green-Blue (RGB) model evaluation for the a) reference, b) direct injection and c) optimized PT-µSPE methods.

**
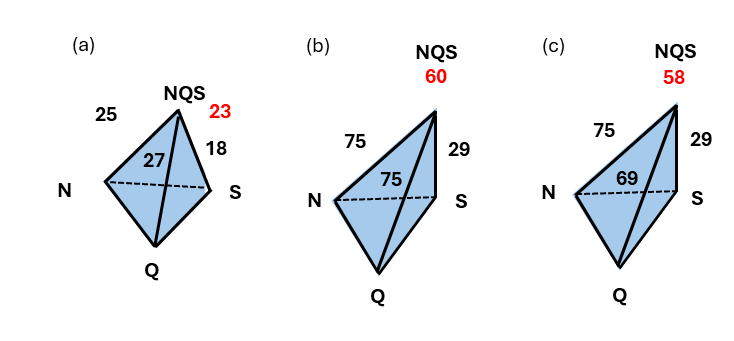
**

**Figure S8.** Comparison of Need Quality Sustainability (NQS) index results for the a) reference, b) direct injection and c) optimized PT-µSPE methods.

**
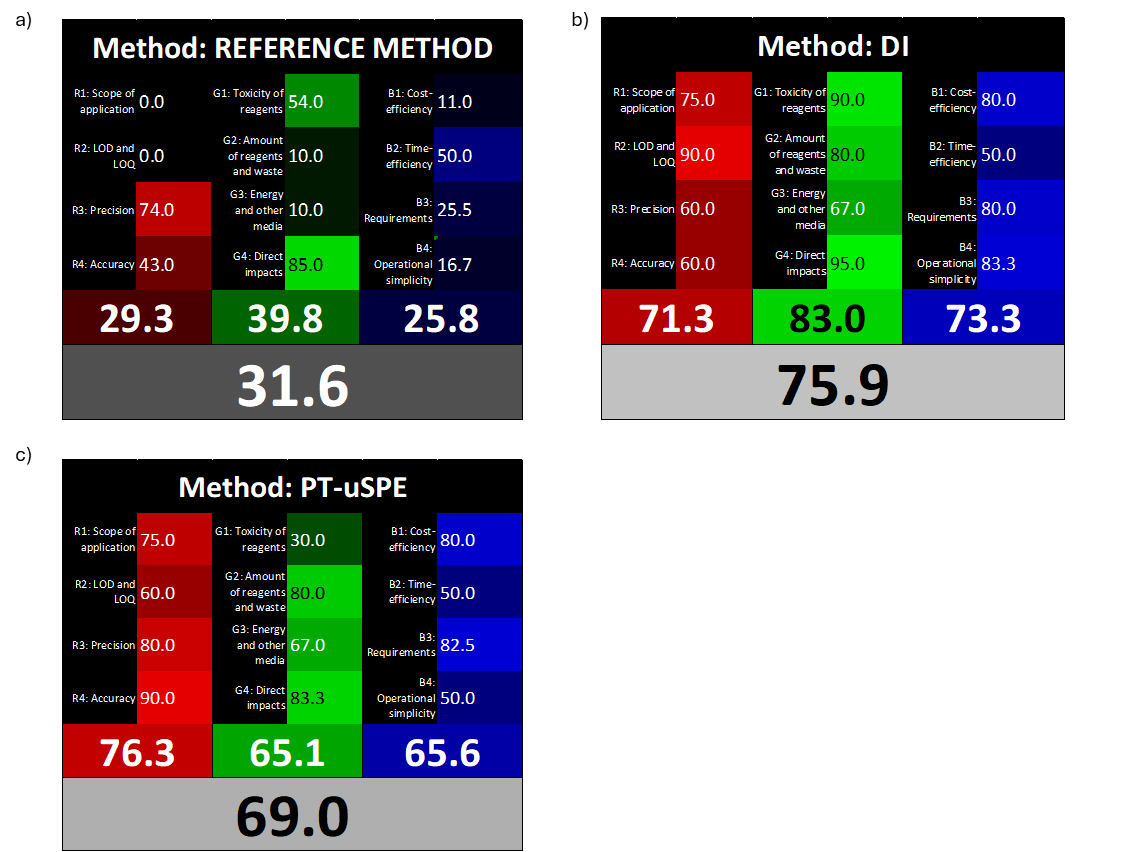
**

**Figure S9.** Comparison of White Analytical Chemistry (WAC) results for the a) reference, b) direct injection and c) optimized PT-µSPE methods.
